# Cross-linked beta alumina nanowires with compact gel polymer electrolyte coating for ultra-stable sodium metal battery

**DOI:** 10.1038/s41467-019-11960-w

**Published:** 2019-09-18

**Authors:** Danni Lei, Yan-Bing He, Huijuan Huang, Yifei Yuan, Guiming Zhong, Qiang Zhao, Xiaoge Hao, Danfeng Zhang, Chen Lai, Siwei Zhang, Jiabin Ma, Yinping Wei, Qipeng Yu, Wei Lv, Yan Yu, Baohua Li, Quan-Hong Yang, Yong Yang, Jun Lu, Feiyu Kang

**Affiliations:** 1 Shenzhen Geim Graphene Center, Tsinghua Shenzhen International Graduate School, Tsinghua University, Shenzhen, 518055 China; 2 State Key Laboratory of Optoelectronic Materials and Technologies, School of Materials Science and Engineering, Sun Yat-sen University, Guangzhou, 510275 China; 3 Hefei National Laboratory for Physical Sciences at the Microscale, Department of Materials Science and Engineering, Key Laboratory of Materials for Energy Conversion, Chinese Academy of Sciences (CAS), University of Science and Technology of China, Hefei, 230026 Anhui China; 4 Chemical Sciences and Engineering Division, Argonne National Laboratory, Lemont, IL 60439 USA; 5 Xiamen Institute of Rare Earth Materials, Haixi institutes, Chinese Academy of Sciences, Xiamen, 361024 China; 6 School of Chemical Engineering and Technology, Xi’an Jiaotong University, Xi’an, 710049 China; 7 Shenzhen Environmental Science and New Energy Technology Engineering Laboratory, Tsinghua-Berkeley Shenzhen Institute (TBSI), Tsinghua University, Shenzhen, 518055 China; 8 Dalian National Laboratory for Clean Energy (DNL), Chinese Academy of Sciences (CAS), Dalian, 116023 Liaoning China; 9 Nanoyang Group, School of Chemical Engineering and Technology, Tianjin University, Tianjin, 300072 China; 10 State Key Laboratory for Physical Chemistry of Solid Surface, Department of Chemistry, College of Chemistry and Chemical Engineering, Xiamen University, Xiamen, 361005 China

**Keywords:** Batteries, Batteries

## Abstract

Sodium metal batteries have potentially high energy densities, but severe sodium-dendrite growth and side reactions prevent their practical applications, especially at high temperatures. Herein, we design an inorganic ionic conductor/gel polymer electrolyte composite, where uniformly cross-linked beta alumina nanowires are compactly coated by a poly(vinylidene fluoride-co-hexafluoropropylene)-based gel polymer electrolyte through their strong molecular interactions. These  beta alumina nanowires combined with the gel polymer layer create dense and homogeneous solid-liquid hybrid sodium-ion transportation channels through and along the nanowires, which promote uniform sodium deposition and formation of a stable and flat solid electrolyte interface on the sodium metal anode. Side reactions between the sodium metal and liquid electrolyte, as well as sodium dendrite formation, are successfully suppressed, especially at 60 °C. The sodium vanadium phosphate/sodium full cells with composite electrolyte exhibit 95.3% and 78.8% capacity retention after 1000 cycles at 1 C at 25 °C and 60 °C, respectively.

## Abstract (web summary)

Here the authors show a beta alumina nanowires/gel polymer composite electrolyte design. The dense and homogeneous solid-liquid hybrid sodium-ion transportation channels promote uniform sodium deposition and stripping and significantly improve the performance of a Na metal battery.

## Introduction

Severe environmental issues and nonrenewable resource shortages have prompted research on high-performance rechargeable batteries for efficient storage of renewable energy from solar and wind sources^1,2^. Commercial lithium-ion batteries (LIBs) require expensive and rare lithium (Li) resources, which greatly restricts the wide application of LIBs in energy storage systems. In recent years, sodium-ion batteries (SIBs) as a promising alternative to LIBs have attracted increasing attention due to the high natural abundance and low cost of Na resources^3–6^. A sodium (Na) metal anode possesses a high theoretical capacity (1166 mAh g^−1^) and low redox potential (−2.71 V vs. the standard hydrogen potential) that results in Na metal batteries with high working voltage and high-energy density. Therefore, there is great interest in developing Na metal batteries^7–9^, such as Na–O_2_ batteries^10,11^, Na–sulfur (Na–S) batteries^7^ and Na–metal halide batteries^12^. However, utilizing Na metal presents huge safety issues, which are even more serious than those with Li metal. First, the Na metal anode suffers from nonuniform Na stripping and platting due to the inhomogenous current distribution on the surface of Na metal. It is desirable yet challenging to construct a uniform and compact solid electrolyte interphase (SEI) on Na metal to effectively passivate the Na metal surface and suppress Na dendrite formation. Second, similar to the Li metal, the Na metal is highly chemically and electrochemically reactive with organic liquid electrolytes, especially at high temperatures. The reactions lead to electrolyte decomposition, which results in the low Coulombic efficiency, poor cycling stability and severe gassing behavior of Na metal batteries^13^. These issues must be addressed to achieve Na metal batteries with long cycling stability and good safety for a wide range of working temperatures.

Some efforts have already been made to suppress Na dendrite growth, including using suitable electrolyte systems that are stable with a Na metal electrode^14–18^, constructing a stable artificial SEI on the Na metal surface to protect the electrode from incessant corrosion^19–24^, and designing 3D hosts with high specific surface area (e.g., porous copper matrix^25^, nitrogen and sulfur co-doped carbon nanotube paper^26^, 3D flexible carbon felt^27^, conductive carbonized wood^28^, and porous Al^29^) to manipulate Na nucleation behavior. Gel polymer electrolytes (GPEs) with a room-temperature ionic conductivity of 10^−3^ S cm^−1^ have been widely applied in Li metal batteries to increase their safety and energy density by avoiding the leakage of electrolytes^30–33^, and they have also been employed in Na metal batteries^14,34^. Poly(methyl methacrylate) (PMMA) and poly(vinylidene fluoride-co-hexafluoropropylene) (PVdF–HFP)-based GPEs are the most studied types of GPEs^35–37^. To further improve the properties of GPEs, researchers have incorporated SiO_2_ additives^38^, commercial glass fiber (GF) membranes^39^, and polydopamine (PDA)^40^ into the above GPEs. However, these additives are nonionic conductors, which inhibit the formation of a stable Na/GPE interface. In addition, the high-temperature stability of Na metal batteries has received less attention. Therefore, it is rather important to develop a homogeneous inorganic ionic conductor/GPE with high-ionic conductivity, smooth surface and compact structure to induce uniform Na stripping and platting and suppress dendrite formation. This composite may provide a new method to achieve stable Na metal batteries at wide working temperatures.

Polycrystalline beta alumina (Al_2_O_3_) with high ionic conductivity and good thermal properties is a solid electrolyte material used in Na–S and Na–nickel chloride batteries that operate at 300–350 °C^41,42^. Beta Al_2_O_3_ has two similar crystalline phases of β-Al_2_O_3_ (NaAl_11_O_17_; hexagonal; P63/mmc; a_0_ = b_0_ = 5.58 Å, c_0_ = 22.45 Å) and β″-Al_2_O_3_ (NaAl_5_O_8_; rhombohedral; R3m; a_0_ = b_0_ = 5.60 Å, c_0_ = 33.95 Å). The β″-phase is more desirable since its ionic conductivity is 3–4 times higher than that of the β-phase.

**Fig. 1:**
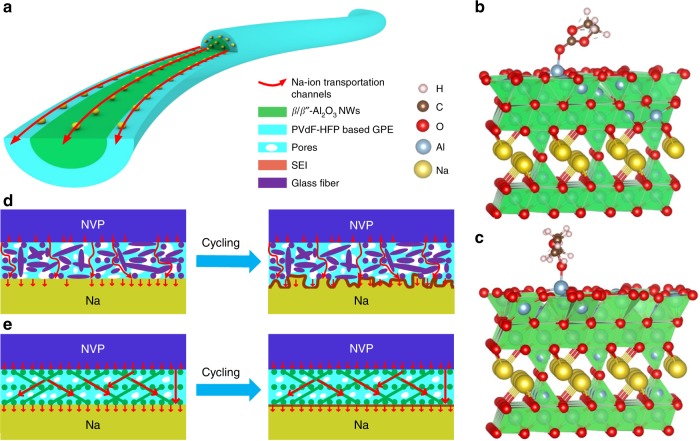
Schematic of NVP/Na batteries using ANs–GPE and GFs-GPE electrolytes. **a** Structure and Na-ion transportation mechanism of ANs–GPE. **b**, **c** Adsorption of ethylene carbonate (EC) and diethyl carbonate (DEC) on β″-Al_2_O_3_ (003). **d**, **e** Working mechanism of NVP/Na batteries during long cycles with GFs-GPE and ANs–GPE. In the NVP/ANs–GPE/Na batteries, the flat ANs–GPE creates dense, uniform and solid–liquid hybrid Na-ion transportation channels on the surface of the Na metal anode that contribute to uniform Na deposition and the formation of stable and smooth SEI films during long cycles, while in the NVP/GFs-GPE/Na batteries, uneven Na deposition occurs due to the nonionic conductive GFs and highly porous structure of GFs-GPE

Here, we develop a hybrid inorganic ionic conductor/GPE composite, in which a cross-linked β/β″-Al_2_O_3_ Nanowires (ANs) membrane with 78.1% β″-phase is compactly coated by PVdF–HFP–based GPE (Fig. 1a). In this innovative structure (ANs–GPE), the cross-linked ANs membrane combined with the PVdF–HFP polymer coating layer can effectively immobilize the liquid electrolyte to construct highly efficient, uniform, and continuous solid–liquid hybrid Na-ion transportation channels through and along the ANs (Fig. 1b, c). The cross-linked ANs membrane as an ionic conductor not only boosts the long-range ion motion but also creates dense and uniform Na-ion transportation channels in the ANs–GPE to induce uniform Na deposition and stripping, which is quite different with that using GFs-GPE (Fig. 1d, e; Supplementary Fig. 1a). As a result, a stable and flat SEI is constructed on the Na metal anode, which greatly improves the Na/ANs–GPE interface compatibility. The side reactions between Na metal anode and liquid electrolyte as well as Na dendrite formation are successfully suppressed. Full cells assembled with the Na_3_V_2_(PO_4_)_3_(NVP) cathode, ANs–GPE electrolyte and Na metal anode present a significant improvement in cycling stability in the temperature range of 25–60 °C. Specifically, its capacity retention after 1000 cycles at 1 C under 25 °C and 60 °C is as high as 95.3% and 78.8%, respectively. To the best of our knowledge, this work first proposes and reveals the solid–liquid hybrid Na-ion transportation channels in hybrid electrolytes containing ceramic electrolyte and GPE. This work also highlights the importance and effectiveness of suitable composite electrolytes with inorganic ionic conductor in stabilizing solid-state Na metal batteries at both room and high temperatures.

## Results

### Characterization of ANs and ANs–PVdF–HFP composite

**Fig. 2:**
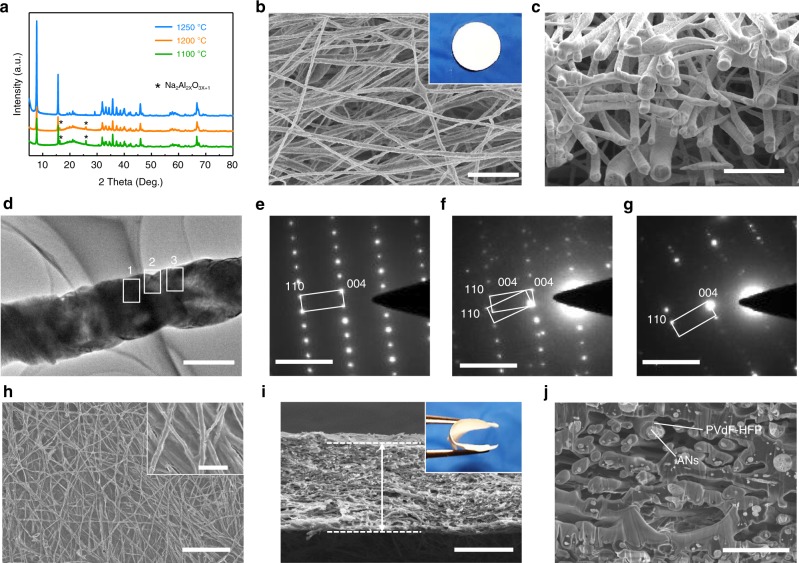
Morphology and structural characterization of ANs and ANs–PVdF–HFP membranes. **a** XRD patterns of ANs prepared at different temperatures. **b** Surface and (**c**) cross-sectional SEM image of the ANs membrane. **d **TEM image of a single AN. **e**, **f**, **g** SAED patterns of areas 1, 2, and 3 in (**d**). **h** Surface and (**i**, **j**) cross-sectional SEM images of ANs–PVdF–HFP membranes. The inset in (**i**) is the digital image of the ANs–PVdF–HFP membrane. Scale bars, 5 μm in **b**, **c**; 500 nm in **d**; 5 1/nm in **e**, **f**, **g**; 25 μm in **h**; 2.5 μm inset in (**h**); 50 μm in **i**; and 10 μm in **j**

The ANs membrane was prepared by a simple electrospinning method and an annealing process. X-ray diffraction (XRD) patterns of the prepared membrane suggest the formation of the hybrid phase of β-Al_2_O_3_ and β″-Al_2_O_3_ after annealing at 1250 °C (Fig. 2a). Since the β-Al_2_O_3_ and β″-Al_2_O_3_ phases have similar X-ray diffraction peaks, the relative amount of the β″-Al_2_O_3_ phase can be calculated using the major peak intensities of XRD patterns of the ANs membrane, as shown in Eq. (1)^43^.

1 $$f\left( {\mathrm{{\beta}}{\prime\prime}} \right)\ = 100\% - f\left( {\mathrm{\beta}} \right)\ = \left( {1 - \frac{{1.14I\left( {\mathrm{\beta}} \right)}}{{1.14I\left( {\mathrm{\beta}} \right) + I\left( {\mathrm{{\beta}}{\prime\prime}} \right)}}} \right) \times 100\%$$

where 1.14 is the I-correction factor, which is related to the relative intensities of the single-phase peaks for β-Al_2_O_3_ and β″-Al_2_O_3_. I_β″_ and I_β_ are the peak intensities at 45.9^o^ and 44.5^o^, respectively. The calculated content of β″-Al_2_O_3_ in the prepared ANs membrane is 78.1%. The density functional theory (DFT) calculations show that the adsorption energies of ethylene carbonate (EC) and diethyl carbonate (DEC) on β″-Al_2_O_3_ (003) are 0.730 and 0.935 eV, respectively (Fig. 1b, c), which are almost two times higher than those on SiO_2_ (001) (0.343 eV and 0.401 eV; Supplementary Fig. 1b, c and Table 1), indicating that the cross-linked ANs membrane can immobilize the liquid electrolyte more effectively than GFs. The scanning electron microscope (SEM) image of Fig. 2b presents that the ANs membrane consists of cross-linked nanowires with an average diameter of ~380 nm (Supplementary Fig. 2), and these nanowires can be cut into small round sections, as shown in the inset of Fig. 2b. The cross-sectional SEM image of the ANs membrane further confirms that the ANs are cross-linked inside the membrane (Fig. 2c), and this cross-linking benefits the transportation of Na ions and enhances the mechanical strength of the ANs–GPE. The transmission electron microscopy (TEM) image shows that the ANs consist of interconnected bamboo-like β/β″-Al_2_O_3_ single crystals (Fig. 2d). The corresponding selective area electron diffraction (SAED) patterns present that the two interconnected grains have the same phase, but different crystal orientations (Fig. 2e–g).

The ANs–PVdF–HFP membrane was produced by soaking the ANs membrane in PVdF–HFP solution and then naturally drying. The microstructural morphologies of the ANs–PVdF–HFP membrane are presented in Fig. 2h–j. The surface SEM images show that the cross-linked ANs are uniformly embedded inside the dense PVdF–HFP matrix (Fig. 2h). The cross-sectional SEM image shows uniform and regular pores inside the ANs–PVdF–HFP membrane (Fig. 2i), and these pores can store the electrolyte to shorten the Na-ion migration path and enhance the ionic conductivity^39^. Figure 2j shows that the ANs are compactly coated and surrounded by PVdF–HFP layer, suggesting an excellent contact and adhesion capability between the ANs and PVdF–HFP, which is ascribed to the higher absorption ability of ANs for PVdF–HFP than GFs (Supplementary Fig. 3). The differential scanning calorimetry (DSC) curves display that the melting temperatures (*T*_m_) decrease with the addition of ANs into the PVdF–HFP copolymers from 145.6 to 140.6 °C, indicating that the degree of crystallinity of PVdF–HFP is reduced because the introduction of ANs can enlarge the amorphous region in the polymer matrix and accelerate dynamic processes with the plasticizing effect (Supplementary Fig. 4a)^44,45^. In addition, the XRD patterns show that the intensity of peaks corresponding to the (020), (110), (022), (200), and (041) planes of γ-phase PVdF greatly decreases (Supplementary Fig. 4b)^44,46^, further confirming the decrease in the crystallinity of PVdF with the addition of ANs. According to the nitrogen adsorption/desorption isotherms, the Brunauer–Emmett–Teller (BET) specific surface area of the ANs–PVdF–HFP membrane is 2.36 m^2^ g^−1^, which is obviously lower than that of the ANs membrane (3.73 m^2^ g^−1^) and PVdF–HFP membrane (6.45 m^2^ g^−1^) (Supplementary Fig. 5a–c). The good compatibility between the ANs and PVdF–HFP results in a relatively dense structure of ANs–PVdF–HFP membrane. The energy dispersive spectroscopy (EDS) results for the surface and cross-sectional of ANs–PVdF–HFP membrane also prove that the ANs network is uniformly dispersed inside the ANs–PVdF–HFP membrane, and the ANs are compactly coated by the PVdF–HFP layer (Supplementary Fig. 6). Thermogravametric analysis (TGA) result presents that the ANs content is ~17 wt% (Supplementary Fig. 7). In contrast, the GFs membrane has larger pores and looser structure compared with the cross-linked ANs, and the PVdF–HFP embedded in GFs membrane has the properties of pure porous PVdF–HFP, which results in the larger surface area of GFs–PVdF–HFP than that of GFs (3.78 vs. 2.25 m^2^ g^−1^, Supplementary Fig. 8a–c, 9a, and 5d, e). The thickness and mass of the ANs–PVdF–HFP membrane is ~80 μm and 11.5 mg (Fig. 2i; Supplementary Table 2), which is much thinner and lighter than that of the GFs–PVdF–HFP membrane (200 μm and 31.1 mg; Supplementary Fig. 8d and Table 2). Therefore, the application of this ANs–PVdF–HFP membrane can significantly enhance the volumetric and gravimetric energy density of cells.

### Electrochemical characterization of ANs–GPE-based cells

**Fig. 3:**
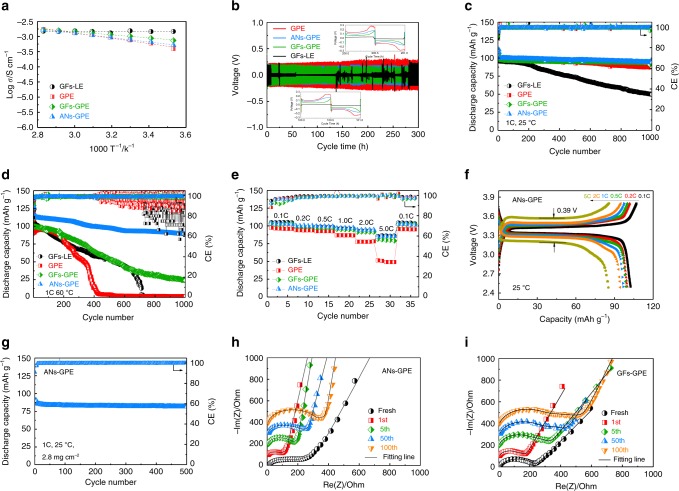
Electrochemical characterizations of ANs–GPE-based cells. **a** Ionic conductivities of GFs–LE, GPE, GFs–GPE, and ANs–GPE. **b** Galvanostatic cycling curves of Na/Na symmetrical cells using GFs–LE, GPE, GFs–GPE, and ANs–GPE at a current density of 0.5 mA cm^−2^. **c**, **d** Long-term cycling performance of NVP/Na cells using GFs–LE, GPE, GFs–GPE, and ANs–GPE at 1 C under 25 °C and 60 °C. **e** Rate performance of NVP/Na cells using GFs–LE, GPE, GFs–GPE, and ANs–GPE. **f** Charge/discharge curves of Na/ANs–GPE/Na symmetrical cells from 0.1 to 5 C. **g** Cycling performance of NVP/ANs–GPE/Na cells with NVP mass loading of 2.8 mg cm^−2^. **h**, **i** EIS plots of NVP/ANs–GPE/Na and NVP/GFs–GPE/Na cells after different cycles

Figure 3a presents that the GFs–LE has the highest ionic conductivity of 1.45 × 10^−3^ S cm^−1^ at 25 °C due to its much larger liquid electrolyte absorption rate than ANs–GPE, GFs–GPE, and GPE (Supplementary Table 2). The ionic conductivity of ANs–GPE is 7.13 × 10^−4^ S cm^−1^ at 25 °C, which is lower than that of GFs–GPE (9.46 × 10^−4^ S cm^−1^) and close to that of PVdF–HFP-based GPE (6.79 × 10^−4^ S cm^−1^). Note that the liquid electrolyte absorption rate of the ANs–PVdF–HFP membrane is obviously lower than that of PVdF–HFP and GFs–PVdF–HFP membranes with an obviously more porous structure and higher surface area (Supplementary Table 2 and Supplementary Fig. 9). Therefore, the cross-linked ANs membrane combined with the PVdF–HFP polymer coating layer can effectively immobilize the liquid electrolyte that may construct highly efficient, uniform and continuous solid–liquid hybrid ionic transportation channels along the ANs. The cross-linked ANs membrane not only boosts the long-range ion motion of polymer branches in the ANs–GPE but also creates dense and uniform Na-ion transportation channels that can improve the ionic conductivity of the whole ANs–GPE membrane. The linear sweep voltammetry (LSV) curves of both ANs–GPE and GFs–LE exhibit no oxidation peak up to 4.8 V vs. Na/Na^+^, which demonstrates the high electrochemical stability of ANs–GPE (Supplementary Fig. 10).

A polarization test of the Na/Na symmetric cells was performed to investigate the dynamic stability of the Na/electrolyte interface (Fig. 3b). Notably, the cells using GPE, GFs–GPE, and ANs–GPE at 0.5 mA cm^−2^ displays a smooth potential with a low overpotential of around 0.2 V from 1 h to 300 h, while the Na/GFs–LE/Na cell displays a short circuit after only 9 h due to the growth of Na dendrites, indicating that the application of GPE can effectively suppress Na dendrite growth. Therefore, the Na/ANs–GPE/Na and Na/GFs–GPE/Na cells present more stable polarization potential than Na/GPE/Na cell at 0.5 mA cm^−2^, suggesting the ANs and GFs can decrease the polarization of Na stripping and platting. The enlarged voltage profiles at 100 h and 200 h in the inset of Fig. 3b present that the Na/GPE/Na symmetric cell shows the largest overpotential and the Na/GFs–GPE/Na cell displays less overpotential than Na/ANs–GPE/Na cell due to the higher ionic conductivity of GFs–GPE. The polarization behavior of Na/Na symmetrical cells using GFs–LE, GPE, GFs–GPE, and ANs–GPE was also examined at a higher current density of 1 mA cm^−2^ (Supplementary Fig. 11a). The Na/Na cells using GFs–LE, GPE, and GFs–GPE exhibit short circuit after 2, 75, and 155 h, respectively, while that using ANs–GPE presents stable polarization potential after 300 h. Furthermore, even at higher current of 2 mA cm^−2^, the Na/ANs–GPE/Na cell can stably cycle for over 100 h, while the Na/GFs–GPE/Na cell displays short circuit only after 43 h (Supplementary Fig. 11b). This result exhibits that the ANs induce the uniform deposition of Na metal for an excellent cycling stability of Na/Na symmetrical cells.

Figure 3c presents the long-term cycling performances of NVP/Na cells using GFs–LE, GPE, GFs–GPE, and ANs–GPE at 25 °C. The discharge capacity of the NVP/GFs–LE/Na cell slightly decreases during the initial 200 cycles at 1 C (the corresponding current density is 0.17 mA cm^−2^), and then sharply decreases (Fig. 3c). Its capacity retention (52.0%) is much smaller than that of the NVP/GPE/Na cell (87.9%) after 1000 cycles. The reason for this difference is that the application of GPE can effectively suppress the growth of Na dendrites and reduce the side reactions of the electrolyte with the Na metal anode due to the absorption ability of the GPE for the liquid electrolyte^37^. Furthermore, the NVP/GFs–GPE/Na and NVP/ANs–GPE/Na cells present more stable cycling performances than NVP/GPE/Na cell and exhibit almost the same capacity retention after 1000 cycles (95.7% for NVP/GFs–GPE/Na and 95.3% for NVP/ANs–GPE/Na). The addition of GFs and ANs membranes in GPE further greatly improve the cycling performance of NVP/Na cells at 25 °C similar to Na/Na symmetric cells. However, the NVP/Na cells using GFs–LE, GPE, GFs–GPE, and ANs–GPE present quite different cycling behaviors at 60 °C (Fig. 3d). Unexpectedly, the NVP/GPE/Na cell fails after only 400 cycles, and the NVP/GFs–LE/Na cell presents relatively good cycling performance but also fails after 700 cycles. The reason for failure is that the GPE becomes soft at 60 °C, which compromises its ability to suppress Na dendrite growth during cycling, resulting in the worst cycle stability. Therefore, there are severe side reactions between the Na metal anode with dendrites and the liquid electrolyte in both the NVP/GPE/Na and NVP/GFs–LE/Na cells at 60 °C.

Intriguingly, the capacity retention of the NVP/ANs–GPE/Na cell after 1000 cycles is up to 78.8%, which is almost three times higher than that of NVP/GFs–GPE/Na cell (24.6%). The NVP/ANs–GPE/Na cell presents quite stable Coulombic efficiency up to 99% during 1000 cycles, which is ascribed to that the ANs–GPE can effectively promotes the uniform Na deposition and the formation of a stable and flat SEI on Na metal anode. As a result, the side reaction of liquid electrolyte in ANs–GPE with Na metal anode can be successfully suppressed. Whereas, the Coulombic efficiency of NVP/Na cells using GFs–LE, GPE, and GFs–GPE fluctuates obviously due to the serious side reactions between Na metal anode and electrolyte at 60 °C, especially for that using GFs–LE and GPE. The (dis)charge overpotential of the NVP/ANs–GPE/Na cell cycled for 500 times is 0.18 V, which is much smaller than that of the NVP/GFs–GPE/Na cell (0.63 V) and NVP/GFs–LE/Na cell (0.64 V) (Supplementary Fig. 12a–c). Such significant improvement in the cycling performance and low polarization indicates that ANs–GPE not only remarkably suppresses the side reactions between the Na metal anode and electrolyte solvents even at 60 °C but also successfully suppresses Na dendrite growth. Although GFs–GPE can effectively suppress the side reactions of the Na metal anode with electrolyte at 25 °C to achieve excellent cycling performance, these side reactions still violently occur at 60 °C due to its highly porous structure.

The rate performance of the NVP/ANs–GPE/Na cell is comparable with that of NVP/GFs–LE/Na, but better than that of NVP/GPE/Na and NVP/GFs–GPE/Na cells (Fig. 3e). The discharge capacity of NVP using ANs–GPE at 5 C is 85.5 mAh g^−1^, which is 83.3% of that at 0.1 C. In addition, the (dis)charge overpotential of the NVP/ANs–GPE/Na cell at 5 C is 0.39 V, which is obviously less than that of the NVP/GFs-GPE/Na cell (0.48 V, Fig. 3f; Supplementary Fig. 12d). The NVP/ANs–GPE/Na cell presents an excellent rate performance and low electrochemical polarization. Notably, the specific capacity of the NVP/ANs–GPE/Na cell only slightly decreases from 99.5 to 85.8 mAh g^−1^ at 1 C and from 94.2 to 67.0 mAh g^−1^ at 2 C as the NVP loading increases from 1 to 2.8 mg cm^−1^, and the capacity retention is as high as 95.2% after 500 cycles at 1 C (the current density is 0.48 mA cm^−2^, Fig. 3g; Supplementary Fig. 12f). The electrochemical impedance spectroscopy (EIS) results of the NVP/ANs–GPE/Na and NVP/GFs–GPE/Na cells after different numbers of cycles were obtained and simulated (Fig. 3h, i; Supplementary Fig. 13). The charge transfer resistance (R_ct_) of the fresh NVP/GFs–GPE/Na cell (235.7 Ohm) is much larger than that of the fresh NVP/ANs–GPE/Na cell (149.8 Ohm). Therefore, the smooth surface of ANs–GPE improves the interfacial contact between the electrolyte and electrodes. After the first cycle, the R_ct_ of the NVP/ANs–GPE/Na cell dramatically decreases to 30.4 Ohm, which is much less than that of the NVP/GFs–GPE/Na cell (63.3 Ohm). Furthermore, the R_SEI_ of NVP/ANs–GPE/Na cell is only one-third that of the NVP/GFs–GPE/Na cell (39.6 Ohm vs. 114.0 Ohm). These results indicate that the dense and smooth surface of ANs–GPE favors uniform Na deposition to form a stable and flat SEI film, which greatly improves the Na/ANs–GPE interface compatibility with the Na metal anode to reduce the R_ct_ and R_SEI_. Therefore, the NVP/ANs–GPE/Na cell presents a better rate performance than the NVP/GFs–GPE/Na. As the cycle number increases, both the R_SEI_ and R_ct_ of the NVP/ANs–GPE/Na cell slightly increase while those of the NVP/GFs–GPE/Na cell dramatically increase, suggesting much higher interfacial and chemical stabilities between the electrolyte and Na metal anode using ANs–GPE than GFs–GPE.

## Discussion

**Fig. 4:**
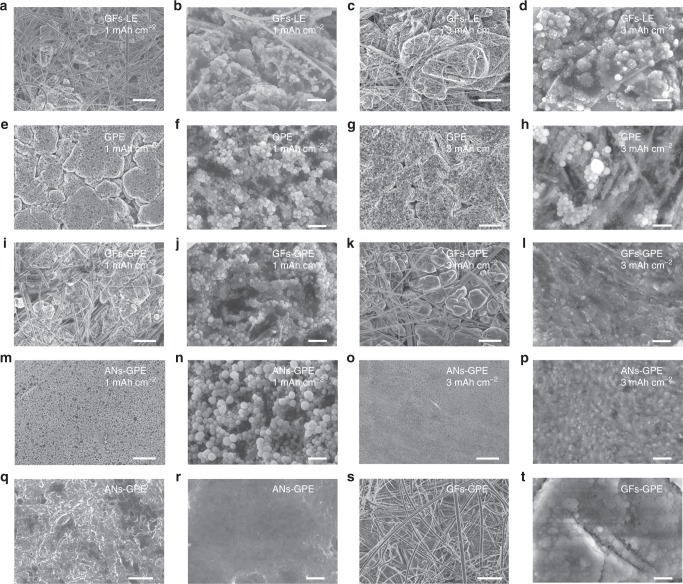
SEM images of Na metal morphology. Areal capacity of 1 and 3 mAh cm^−2^ Na metal was deposited on a Cu surface using GFs–LE (**a**, **b** and **c**, **d**), GPE (**e**, **f** and **g**, **h**), GFs–GPE (**i**, **j** and **k**, **l**) and ANs–GPE (**m**, **n** and **o**, **p**) at current densities of 0.5 mA cm^−2^ and 60 °C. The Cu/Na cells using (**q**, **r**) ANs–GPE and (**s**, **t**) GFs–GPE were stored at 60 °C for 10 days. The SEM images of Fig. 4b, d, f, h, j, l, n, p, r, t are magnified images of Fig. 4a, c, e, g, i, k, m, o, q, s. Scale bars, 10 μm in **a**, **c**, **e**, **g**, **i**, **k**, **m**, **o**, **q**, **s**; and 500 nm in **b**, **d**, **f**, **h**, **j**, **l**, **n**, **p**, **r**, **t**

To identify the Na deposition behavior induced by different electrolytes at high temperature, Cu/Na cells using GFs–LE, GPE, GFs–GPE, and ANs–GPE were assembled, and areal capacities of 1 and 3 mAh cm^−2^ Na metal were deposited on the Cu surface at a current density of 0.5 mA cm^−2^ at 60 °C. The Na deposition layer using GFs –LE is extremely heterogeneous, and most of the Na metal was deposited inside the pores of GFs, which would greatly increase the surface area of Na metal and result in severe side reaction with electrolyte (Fig. 4a–d). As a result, the Na/GFs–LE/Na cell displays short circuit after only 9 h cycling and NVP/GFs–LE/Na cell presents very poor cycling stability at both 25 and 60 °C. When the GPE is applied, the Na deposition layer has disconnected cracks and consists of nanospheres (Fig. 4e, f). Furthermore, when the deposition content increases from 1 to 3 mAh cm^−2^, some Na metal sheets similar to dendrites form (Fig. 4g, h). Since liquid electrolytes are mostly trapped in pores of GPE, this leads to the enrichment of Na ions on the Cu foil surface facing the pores of the GPE, but there is a lack of Na-ions on the Cu foil surface contacting the skeletons of the GPE^47^. Thus, GPE with an uneven pore size and pore distribution also cannot induce uniform Na deposition at 60 °C, which promotes drastic side reactions between the Na metal anode and electrolyte, resulting in the quite poor cycling performance of the NVP/GPE/Na cell at high temperature (Fig. 3d).

Similar to using GFs–LE, the Na metal using GFs–GPE is also deposited inside the pores of GFs due to the pore structure of GFs–PVdF–HFP membranes (Fig. 4i–l), which results in the unsatisfactory cycling performance of NVP/GFs–GPE/Na cell at 60 °C. On the contrary, when the ANs–GPE was applied, the Na deposition layer on the surface of Cu foil presents a considerably uniform morphology consisting of Na nanospheres, which is quite different from the Na morphology obtained using GFs–LE, GPE, and GFs–GPE (Fig. 4m, n). Interestingly, with an increase in the deposition content to 3 mAh cm^−2^, a more uniform and compact deposition layer forms (Fig. 4o, p). In addition, using similar liquid electrolyte uptake (40 μL) and thickness (80 μm) of GF–LE, GPE, and GFs–GPE with that of ANs–GPE, many large aggregated particles consisting of nanoparticles were also formed on the surface of Cu (Supplementary Figs. 14, 15). So, even with similar liquid electrolyte uptake, thickness, higherionic conductivity (Supplementary Fig. 16a) and larger surface area (Supplementary Fig. 5b–e; Fig. 16c), the GFs–LE, GPE, and GFs–GPE still cannot induce the uniform Na transportation to achieve flat Na deposition as ANs–GPE does due to their heterogeneous Na-ion distribution. In addition, Na deposition is also quite inhomogeneous on the Cu surface using cross-linked nonionic conductor γ-Al_2_O_3_ nanowires–GPE with similar structure to ANs–GPE even with an obvious higher ionic conductivity of 1.07 × 10^−3^ S cm^−1^ at 25 °C (Supplementary Figs. 17–19), indicating that the cross-linked γ-Al_2_O_3_ nanowires with large surface area (70.18 m^2^ g^−1^) also cannot promote the uniform Na metal deposition. We examined the Na-ion transference number (t_Na_^+^) of GFs–LE, GPE, GFs–GPE, ANs–GPE, and γ-Al_2_O_3_–GPE with same liquid electrolyte uptake (40 μL) and thickness (80 μm) via the method proposed by Evans et al.^48,49^ and their values are 0.24, 0.21, 0.29, 0.37, and 0.20, respectively (Supplementary Fig. 20). The ANs–GPE presents obvious higher t_Na_^+^ than other electrolytes. The much higher t_Na_^+^ of ANs–GPE suggests that the ANs as a single-ion conductor promotes the Na-ion transport in ANs–GPE to achieve higher and more uniform Na-ion concentrations at the interface between ANs–GPE and Na metal anode. Above results reinforce our conclusion that the ANs promote more uniform Na-ion transport on the Cu electrode.

**Fig. 5:**
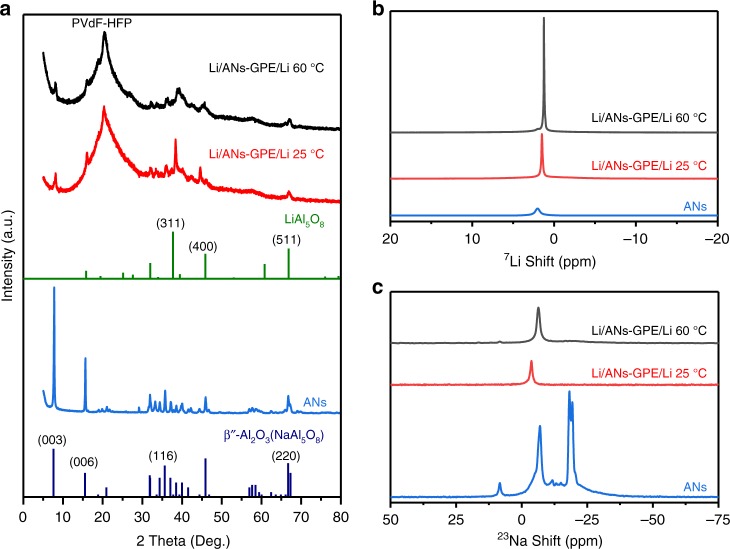
Characterization of ANs before and after cycling in Li/ANs–GPE/Li cells. **a** XRD patterns and (**b**) ^7^Li and (**c**) ^23^Na NMR spectra of ANs and cycled ANs–GPE in Li/ANs–GPE/Li cells at 25 °C and 60 °C

In order to reveal the role of networked ANs in ANs–GPE for Na-ion transportation behavior, we assembled the Li/ANs–GPE/Li symmetrical cells and then charged/discharged these cells for 2 h and ten times at a current density of 0.5 mA cm^−2^ at 25 °C and 60 °C, respectively. Then, we examined the XRD patterns, the ^7^Li and ^23^Na nuclear magnetic resonance (NMR) of ANs before and after cycling to analyze the substitution behavior between Na ion and Li ion to identify and understand the Na-ion transportation of ANs in ANs–GPE. XRD data in Fig. 5a and Supplementary Fig. 4b present that a new and main phase of LiAl_5_O_8_ is formed in the cycled ANs–PVdF–HFP membrane, indicating that most of the Na ions in the ANs are substituted by the Li ions. The ^7^Li and ^23^Na NMR spectra of ANs and cycled ANs–PVdF–HFP membrane in Li/ANs–GPE/Li cells present that the intensity of ^7^Li signal increases apparently after cycling, while the ^23^Na signal decreases greatly compared with that of the original ANs (Fig. 5b, c). This also indicates that the most of Na ions in ANs are replaced by the Li ions. Both of the XRD and NMR results prove that the Na ions in ANs are de-intercalated, and the ANs take part in ionic transportation during charge/discharge of ANs–GPE based Na metal batteries to form solid–liquid hybrid Na-ion transportation channels through and along the ANs in the ANs–GPE. ANs–GPE with dense and uniform Na-ion transportation channels as well as smooth surface acts as a redistributor to regulate Na-ion distribution which are beneficial for even Na-ion flux to achieve homogeneous Na deposition^34,47^. Thanks to the above advantage, the Na dendrite growth and the side reactions between the ANs–GPE and the Na metal anode are successfully suppressed, which contributes to the excellent cycling performance of the NVP/ANs–GPE/Na cell at 60 °C. Furthermore, the Cu/Na cells using GFs–GPE and ANs–GPE were stored for 10 days at 60 °C to further examine the reactivity between the Na metal anode and the electrolyte (Fig. 4q–t). A smooth surface is maintained using ANs–GPE (Fig. 4q, r). In contrast, a very thick and lumpy layer forms when using GFs–GPE (Fig. 4s, t) due to the products from the side reactions between the Na anode and the electrolyte. These results indicate that the ANs–GPE could produce an ultra-uniform Na deposition layer on the Na anode and effectively prevent the continuous side reactions between the Na metal anode and electrolyte at high temperature.

**Fig. 6:**
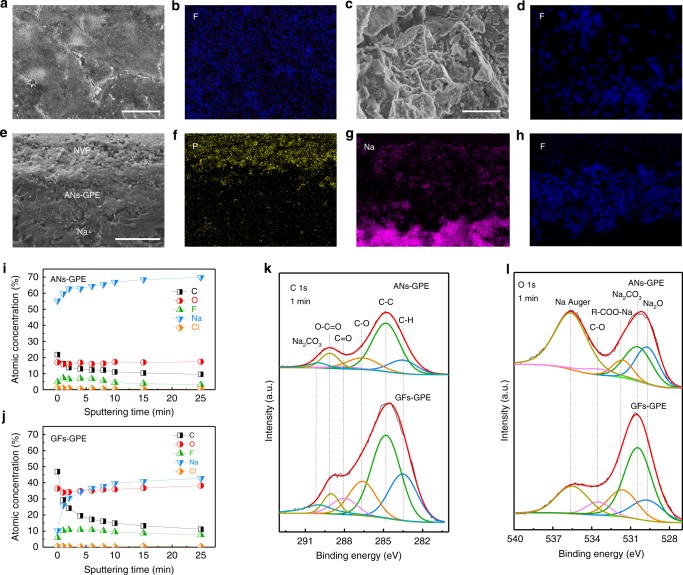
Morphology and components analysis of the Na metal anode after cycling at 1 C using ANs–GPE and GFs–GPE at 60 °C. **a**–**d** SEM and EDS images of the Na metal anode surface after 200 cycles. **e**–**h** SEM and EDS images of cross-section of the NVP/ANs–GPE/Na cell after 1000 cycles. **i, j** Atomic concentration of various elements with sputtering time. **k**, **l** High-resolution C1s and O1s XPS spectra of Na anodes after 200 cycles. Scale bars, 5 μm in **a**, **c**; and 50 μm in **e**

Since the reversibility of Na metal anodes is largely dependent on the composition and morphology of the SEI^50^, SEM, EDS, and X-ray photoelectron spectroscopy (XPS) with depth profiling were carried out to examine the SEI formed on the Na anodes in the NVP/ANs–GPE/Na and NVP/GFs–GPE/Na cells. The Na/NVP cells were disassembled in the fully discharged state after 200 cycles at 60 °C. Figure 6a shows that the surface of the Na anode cycled in the NVP/ANs–GPE/Na cell is quite flat and that the SEI film is considerably uniform, which is further confirmed by the homogeneous distribution of F element derived from the decomposition of FEC (Fig. 6b). In contrast, many Na dendrites form on the surface of the Na metal anodes cycled in the NVP/GFs–LE/Na and NVP/GFs–GPE/Na cells, leading to the formation of a cracked SEI film (Fig. 6c; Supplementary Fig. 21a) and a heterogeneous distribution of F element (Fig. 6d; Supplementary Fig. 21b). Figure 6e–h presents the cross-sectional SEM images and EDS maps of the NVP/ANs–GPE/Na cell after 1000 cycles, and very tight NVP/ANs–GPE and ANs–GPE/Na interfaces are observed. This result clearly confirms that the ANs–GPE has an excellent contact and affinity with both NVP cathode and Na metal anode, which greatly reduce their interfacial resistance. In addition, its smooth surface promotes the formation of a flat interface between ANs–GPE and Na metal anode. The thin and homodispersed liquid electrolyte layer at the interface decomposes to form a flat SEI film. The thickness of such a uniform SEI layer does not obviously increase with cycling due to the uniform Na metal deposition/stripping resulted from the highly efficient and homogeneous solid–liquid hybrid ionic transportation channels of ANs–GPE. Moreover, the ANs–GPE also helps form a thin SEI layer on the surface of the NVP side, and the morphology is very similar to that of the fresh NVP electrode, even after 1000 cycles (Supplementary Fig. 22a, b). However, GFs–LE and GFs–GPE with porous structure brings the heterogeneity of ion distribution and uncontrollable decomposition of electrolyte, leading to the formation of Na dendrites and cracked SEI film on the Na metal anode as well as thick SEI film on the NVP electrode (Supplementary Fig. 22c, d). The corresponding XRD patterns of the NVP electrodes in the discharge state with different electrolytes are similar, indicating no obvious changes in the crystallinity of NVP after 1000 cycles (Supplementary Fig. 23).

We analyzed the composition of the SEI formed on the Na metal anodes using ANs–GPE and GFs–GPE by XPS spectra (Fig. 6i–l; Supplementary Fig. 24). The C, O, F, Na, and Cl contents on the Na metal anode after sputtering for different time were calculated as shown in Fig. 6i, j. The contents of C, O, and F using GFs–GPE are higher than those obtained using ANs–GPE, indicating that the SEI using GFs–GPE contains more elements of C, O, and F. In addition, the contents of C, O, F, and Na using ANs–GPE become stable after only sputtering for 2 min, which is much less than that using GFs–GPE (6 min). Thus, the SEI film that forms on the Na metal anode using ANs–GPE is considerably thinner than that formed using GFs–GPE. In the high-resolution C1s XPS spectra of all the electrodes (Fig. 6k), the peaks corresponding to the species containing C–H (283.3 eV), C–C (284.8 eV), C–O (286.6 eV), C = O (288.0 eV), ROCO_2_Na (289.1 eV), and Na_2_CO_3_ (290.0 eV) were observed, which are the main components of SEI from the decomposition products of the carbonate electrolyte^51–53^. The high-resolution O1s XPS spectra confirm that the SEI contains the species of Na_2_O (529.7 eV), Na_2_CO_3_ (530.4 eV), R-COO-Na (531.6 eV), and C–O (533.5 eV) (Fig. 6l)^51,54^. The XPS peak at 535.5 eV is assigned to Na Auger^54^. The XPS peak intensities of the C1s and O1s spectra of the Na anode using ANs–GPE are much lower than those of the anode in the GFs–GPE-based cells, especially for the peak of Na_2_CO_3_, suggesting that the ANs–GPE effectively mitigates the decomposition of electrolyte^51^. The F1s XPS spectrum presents a single peak at 683.9 eV using ANs–GPE, which is assigned to NaF (Supplementary Fig. 24)^55^. In contrast, the F1s spectrum for the GFs–GPE-based cell shows two peaks at 683.7 eV^55^ and 685.9 eV^56^, respectively, which are assigned to NaF and C–F species as the main reduction product of FEC. It is worth mentioning that the SEI composition on the cathodes is similar to that on the anodes (Supplementary Fig. 25). Therefore, the ANs–GPE effectively immobilize the liquid electrolyte and presents dense, uniform and highly efficient solid–liquid hybrid Na-ion transportation channels to induce uniform Na metal deposition/stripping at the interface between the ANs–GPE and the Na metal anode, which successfully suppresses the Na dendrite growth and continuous side reactions of both the Na metal anode and NVP cathode with electrolyte at high temperature.

In summary, we have developed an inorganic ionic conductor/GPE composite that combines cross-linked ANs membrane and PVdF–HFP. The uniform distribution of ANs membrane in PVdF–HFP can greatly enhance the density and uniformity of solid–liquid hybrid Na-ion transportation channel for homogenous Na deposition and SEI formation. The side reactions between the Na metal anode and the liquid electrolyte as well as Na dendrite formation were successfully suppressed due to the effective immobilization of electrolyte by ANs–GPE and formation of a stable and flat SEI on the Na metal anode. The NVP/ANs–GPE/Na cell displays an excellent capacity retention 78.8% of after 1000 cycles at 1 C and 60 °C. This work first proposes and proves the solid–liquid hybrid Na-ion transportation channels in hybrid electrolytes containing ceramic electrolyte and GPE. This work provides a general strategy to achieve uniform Na metal deposition for long life Na metal battery technology more competitive for energy storage applications, and this strategy may be also suitable for Li, potassium, or other metal batteries.

## Methods

### Materials

Al(NO_3_)_3_·9H_2_O (99%) and C_2_H_4_O_2_ (99.5%) were purchased from Macklin. NaNO_3_ (99%) were obtained from Damao Chemical Reagent Factory. HNO_3_ (65%-68%) were purchased from Guangzhou Chemical Reagent Factory. Polyvinyl pyrrolidone (PVP, Mw = 1,300,000), PVdF–HFP (Mw = 400,000), and GFs came from Sigma Aldrich. Al(C_3_H_7_OH)_3_ (99.99%) and LiNO_3_ (99%) were purchased from Aladdin. Liquid electrolyte (1 M NaClO_4_ in ethylene carbonate (EC) and diethyl carbonate (DEC) with 5% fluoroethylene carbonate (FEC)) was obtained from Suzhou Qianmin Chemical Reagent Company.

### Preparation of ANs membrane

The ANs membranes were synthesized via an electrospinning method and following an annealing process. In a typical procedure, 8 mmol of Al(NO_3_)_3_·9H_2_O, 1.4 mmol of LiNO_3_, and 9 mmol of NaNO_3_ were dissolved in 9.2 mL of deionized (DI) water by magnetic stirring at room temperature. Then, 2.9 mL of HNO_3_ and 1.9 mL of C_2_H_4_O_2_ were added into the above solution. After that, 40 mmol of Al(C_3_H_7_OH)_3_ was added and stirred for 24 h. Next, 0.36 g of PVP was added and stirred 12 h to obtain a homogeneous stable spinning sol, which was then electrospun into ANs membrane precursors under the electrospinning voltage of 18 kV and pumping rate of 1 mL h^−1^. The obtained nanowires membranes were dried in a vacuum oven for 12 h and then heat treated in a muffle furnace at 600 °C for 1 h with the heating rate of 1 °C min^−1^. At last, the membrane was further heated up to respective 1100, 1200, and 1250 °C for 1 h with the heating rate of 5 °C min^−1^ to obtain the β/β″-Al_2_O_3_ nanowires (ANs) membrane. The γ-Al_2_O_3_ membranes were prepared using the similar method and the details are shown in Supplementary Methods.

### Preparation of ANs–GPE

PVdF–HFP solutions were prepared by dissolving 0.75 g of PVdF–HFP in a solution with 13.5 g of acetone and 0.75 g of ethanol by magnetic stirring at 50 °C for 2 h. The transparent solution was coated onto the ANs membrane by a dip-coating process at 25 °C and then naturally dried. As a comparison, the GFs–PVdF–HFP and γ-Al_2_O_3_–PVdF–HFP composite membranes were prepared by coating the PVdF–HFP solutions onto the GFs membrane and γ-Al_2_O_3_ nanowire membrane using the same method. The pure PVdF–HFP membrane was prepared by adding the PVdF–HFP solutions into a glass dish and then naturally dried. The membranes were immersed in liquid electrolyte (1 M NaClO_4_ in ethylene carbonate (EC) and diethyl carbonate(DEC) with 5% fluoroethylene carbonate (FEC)) for 12 h in an argon-filled glove box (M. Braun) to obtain the ANs–GPE, GFs–GPE, γ-Al_2_O_3_–GPE, and GPE for the battery assemblies and other characterizations.

### Fabrication of cells

The NVP cathode material was synthesized by a simple method according to a previous work^57^. The phase and morphology of NVP were characterized by XRD, Raman, SEM, and TEM analyses (Supplementary Fig. 26). Pure carbon-coated NVP with a flower-like morphology was synthesized. The cathode was prepared by mixing NVP particles, super P, and PVdF in a weight ratio of 7:2:1 by NMP, then casting the slurry on Al foil. After drying at 80 °C, the cathodes with NVP loading of ~1 and 2.8 mg cm^−2^ particles were obtained. CR2032 coin cells were assembled using Na foil as a anode, NVP as a cathode, and ANs–GPE, GFs–GPE, GPE, or GFs–LE as an electrolyte in a glove box.

### Materials characterization

The X-ray diffraction (XRD) measurement of the samples was characterized on a Bruker D8 Advance with Cu-Kα radiation. Their morphologies and structures were analyzed by a scanning electron microscope (SEM, HITACH S4800) with energy-dispersive spectroscopy (EDS) for elemental analysis and a field emission transmission electron microscope (FE-TEM, FEI Tecnai F30). X-ray photoelectron spectroscopy (XPS) measurement was collected on a PHI 5000 VersaProbe II instrument, and depth profiling was obtained using Ar gas cluster ion beam (Ar-GCIB) sputtering at 15 kV and 35 nA over a 2 × 2 mm area. The solid-state ^7^Li and ^23^Na nuclear magnetic resonance (NMR) were performed in a 9.4 T magnetic field with a Bruker 400 MHz AVANCE III spectrometer. The ANs and cycled ANs–PVdF–HFP membrane are filled into 4 mm rotors. The solid-state ^7^Li and ^23^Na magic angle spinning (MAS) NMR spectra are acquired using single pulse under the spinning frequency of 12 kHz. The specific surface areas were characterized on a micromeritics ASAP 2020 apparatus with the BET method. Raman spectra were recorded by a LabRAM HR800 spectrometer using 532 incident radiation. Thermogravametric analysis (TGA) was performed using a Netzsch STA 449F3 thermal analyzer from room temperature to 800 °C at a heating rate of 10 °C min^−1^ in air atmosphere. The differential scanning calorimetry (DSC) measurements were performed on the above thermal analyzer at a rate of 10 °C min^−1^ in the 30–300 °C temperature range under N_2_ atmosphere. The amount of liquid electrolyte uptake (*η*) was measured by soaking slices in liquid electrolyte for 12 h, and recording the weight after wiping excess liquid electrolyte off with filter papers. Then, *η* was calculated using the following equation:

2 $$\eta = \frac{{W_t - W_0}}{{W_0}} \times 100\%$$

Where *W*_0_ is the weight of the dry membrane and *W*_t_ is the weight of the membrane after soaking in liquid electrolyte.

### Electrochemical measurements

The ionic conductivity of ANs–GPE, GFs–GPE, γ-Al_2_O_3_–GPE, GPE, and GFs–LE was measured by EIS from 10^6^ to 10^2^ Hz with a 5 mV AC oscillation on a VMP3 multichannel electrochemical station (Bio Logic Science Instruments, France). The test cells were assembled by a small piece of ANs–GPE, GFs–GPE, γ-Al_2_O_3_–GPE, GPE, or GFs–LE slice sandwiched between two stainless-steel blocking electrodes. Prior to the EIS measurements, the cells were kept at each test temperature (from 10 to 80 °C) for 1 h to reach the thermal equilibrium. The LSV curves were examined from the open-circuit voltage to 5.5 V versus Na/Na^+^ at a scanning rate of 1 mV s^−1^ using a VMP3 multichannel electrochemical station. Galvanostatic charge/discharge tests of cells were performed on a battery test system (LAND CT2001A) with a voltage range from 2.5 to 3.8 V at 25 °C and 60 °C. The EIS of cells cycled for different times was examined using the VMP3 multichannel electrochemical station in the frequency range of 10^−2^–10^5^ Hz by applying a 5 mV AC oscillation. The cycled cells were transferred into a glove box and dissembled for further examination. The Na metal anodes were repeatedly rinsed with DMC and vacuum dried for 12 h to remove residual solvent. The Na metal anodes were transferred into a chamber with a sealed Ar-filled vessel for SEM and XPS examination. Na/Na symmetric cells were assembled and charged/discharged for 0.5 h at a current densities of 0.5, 1, and 2 mA cm^−2^. The Li/ANs–GPE/Li symmetrical cells were assembled and then charged/discharged these cells for 2 h and ten times with a current density of 0.5 mA cm^−2^ at 25 °C and 60 °C, respectively. The test method of the Na-ion transference number (t_Na_^+^) of ANs–GPE, GFs–GPE, γ-Al_2_O_3_–GPE, GPE, and GFs–LE is shown in Supplementary Methods.

### Computational details

The calculations were performed by using the projector-augmented wave method (PAW) within the framework of density functional theory (DFT) as implemented in the Vienna ab initio Simulation Package (VASP). The interaction between electrons was treated by the exchange-correlation functional of the generalized gradient approximation (GGA) with Perdew–Burke–Ernzerh (PBE) parametrization. We used potentials with electronic configurations of Si (3 s^2^3p^2^), Al (3s^2^3p^1^), Na (3s), O (2s^2^2p^4^), C (2s^2^2p^2^), and H (1 s). The cutoff energy of the plane wave basis was set to 450 eV. Both the slabs of Si (001) and NaAl_5_O_8_ (003) were modeled in a p(3 × 3), in which the terminal exposed surface was constructed with O atoms. A Monkhorst–Pack scheme of 5 × 5 × 1 k-meshes was employed for both slabs, of which a vacuum of 20 Å was applied to prevent spurious interaction with periodic images. Calculations stopped when the accuracies reached the required convergence criteria of 1 × 10^−2^ eV Å^−1^ for the residual forces on ions and 1 × 10^−4^ eV for the total energy difference in the electronic self-consistent loop.

The binding strength of EC (DEC and PVdF–HFP monomer) on the surface of the SiO_2_ (001) (or NaAl_5_O_8_ (003)(β″-Al_2_O_3_)) slab can be calculated by the following equation:

3 $$E_{{\mathrm{be}}} = E_{{\mathrm{s,a}}} - E_{\mathrm{s}} - E_{\mathrm{a}}$$

where *E*_be_ is the binding energy of EC on the SiO_2_ (001), *E*_s,a_ is the total energy of the SiO_2_ (001) slab with adsorbed EC, *E*_s_ is the total energy of a clean SiO_2_(001) slab without EC, and *E*_a_ is the energy of EC.

## Supplementary information

Supplementary Information

## Data Availability

The authors declare that all the relevant data are available within the paper and its Supplementary Information file or from the corresponding author upon reasonable request.
