# Supplementary material for: Cross-linked beta alumina nanowires with compact gel polymer electrolyte coating for ultra-stable sodium metal battery
Source: Nat Commun. 2019 Sep 18;10:4244. doi: 10.1038/s41467-019-11960-w (PMC6751212; doi:10.1038/s41467-019-11960-w)
Supplement: Supplementary file 1 — Supplementary Information [file 41467_2019_11960_MOESM1_ESM.pdf]

Supplementary Information

Cross-linked beta alumina nanowires with a compact gel polymer electrolyte coating for ultra-stable sodium-metal battery

Lei et al.

Supplementary Figures

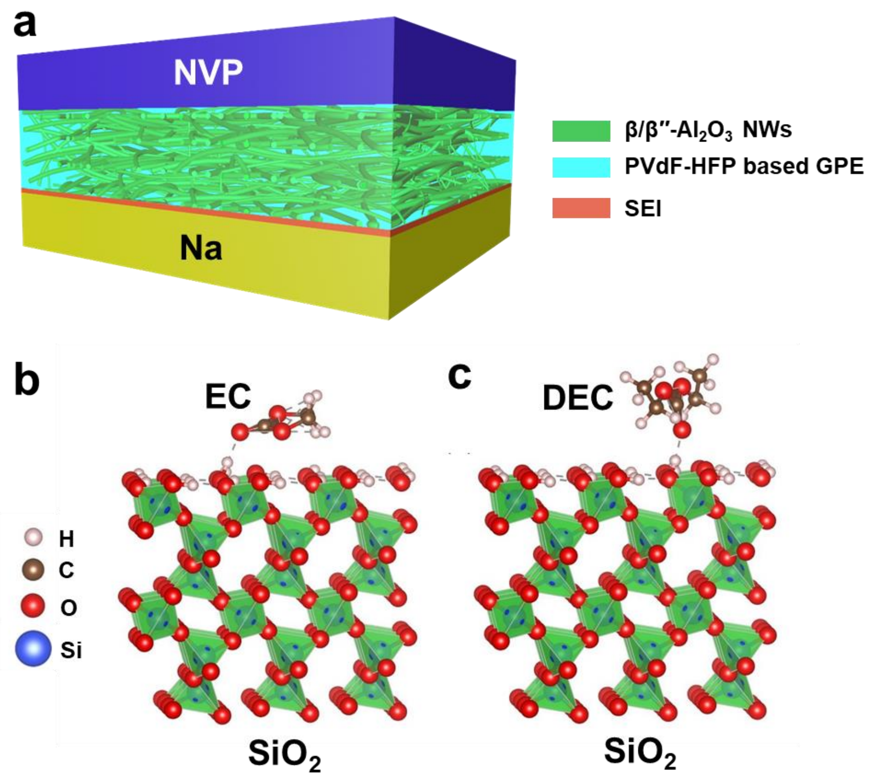

Supplementary Figure 1 Schematic of $Na_3V_2(PO_4)_3$(NVP)/Na batteries and DFT simulation of adsorption. (**a**) NVP/Na battery with ANs-GPE. Adsorption of (**b**) EC and (**c**) DEC on the SiO_2 (001).

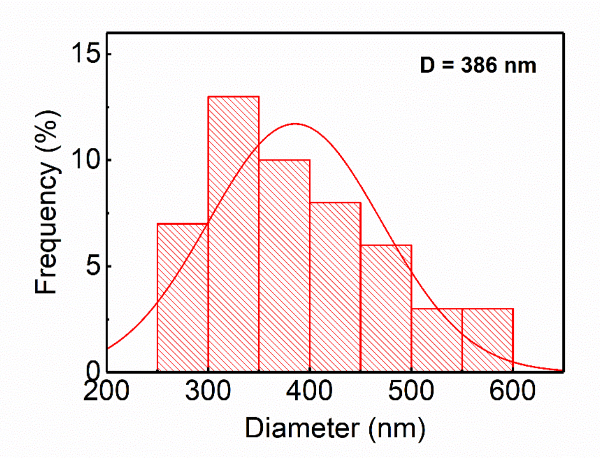

Supplementary Figure 2 Diameter distribution of ANs.

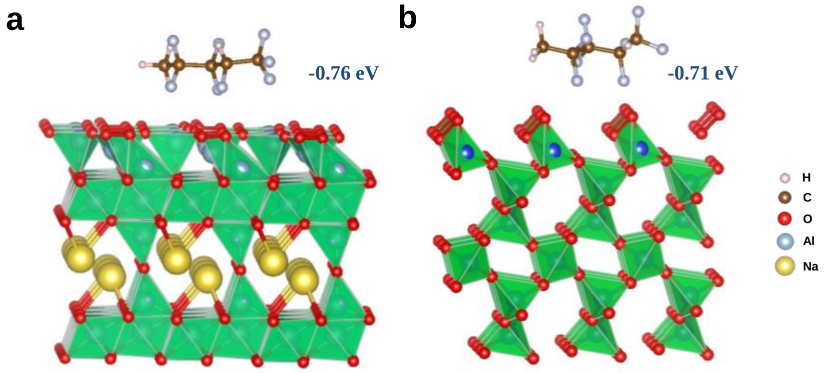

Supplementary Figure 3 DFT simulation of adsorption. The adsorption energies of PVdF-HFP monomer on the (**a**) β″-Al_2O_3(003) and (**b**) SiO_2 (001).

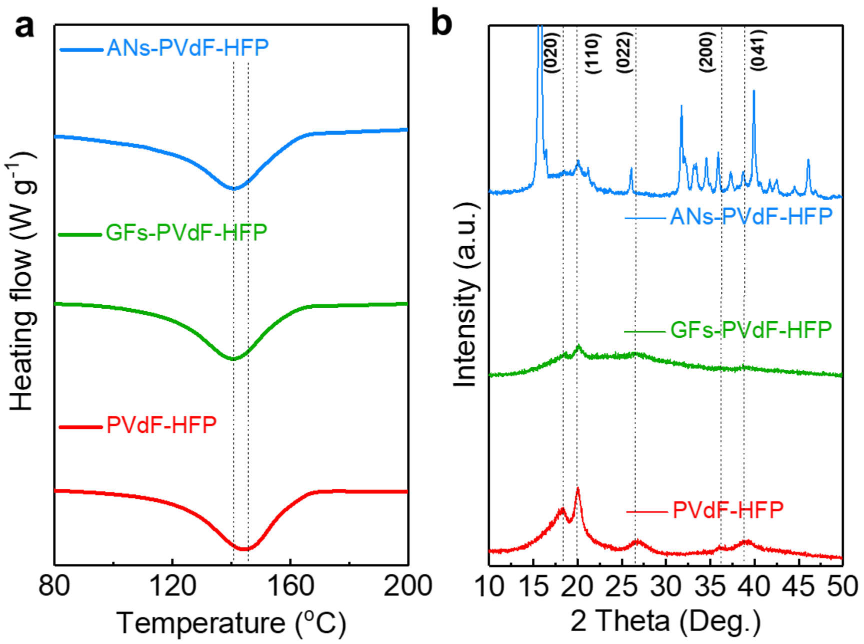

Supplementary Figure 4 Characterization of the crystallinity of different membranes. (**a**) DSC curves and (**b**) XRD patterns of PVdF-HFP, GFs-PVdF-HFP and ANs-PVdF-HFP membranes.

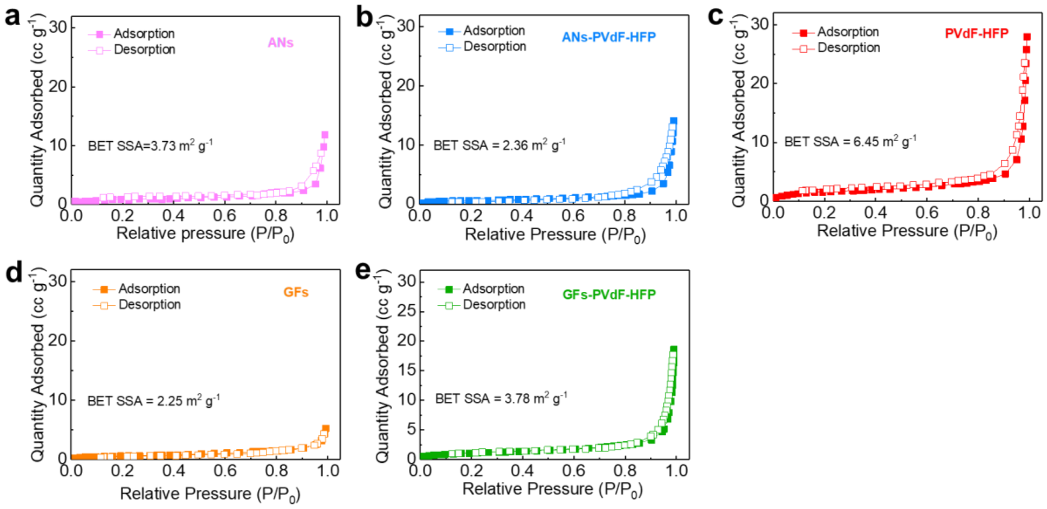

Supplementary Figure 5 Nitrogen adsorption/desorption isotherms of different membranes. (**a**) ANs, (**b**) ANs- PVdF-HFP, (**c**) PVdF-HFP, (**d**) GFs and (**e**) GFs-PVdF-HFP.

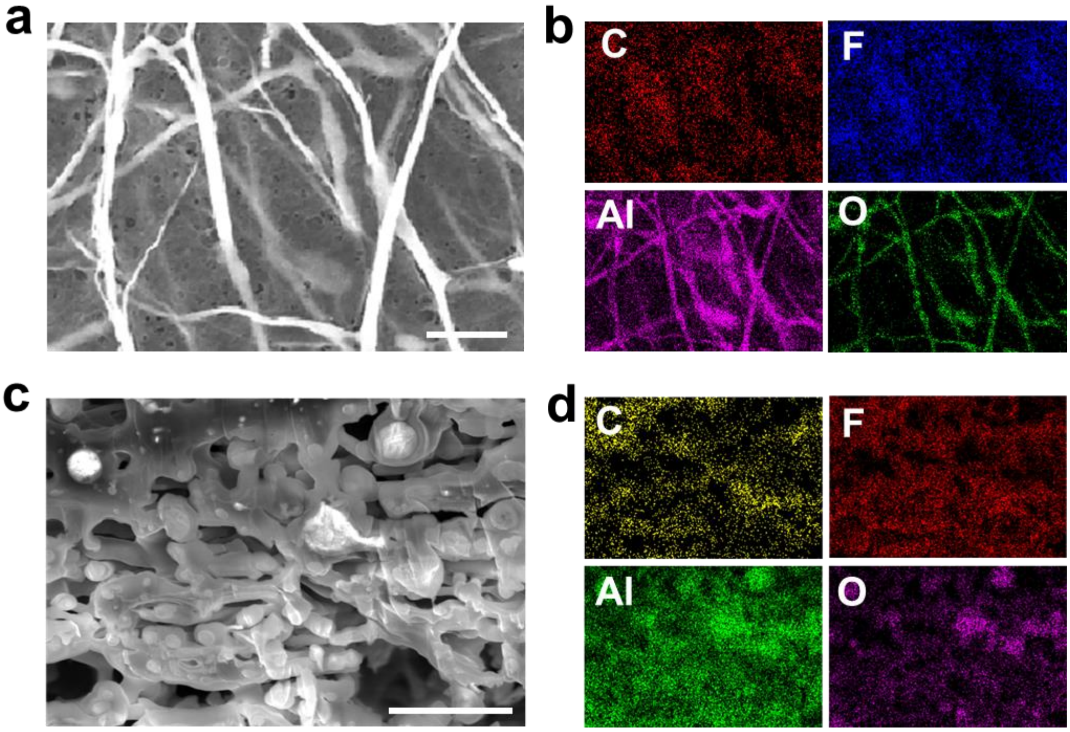

Supplementary Figure 6 SEM and EDS images of ANs-PVdF-HFP membrane. (**a**-**b**) top view, (**c**-**d**) cross section. Scale bars, 2 µm in **a**; 10 µm in **c**.

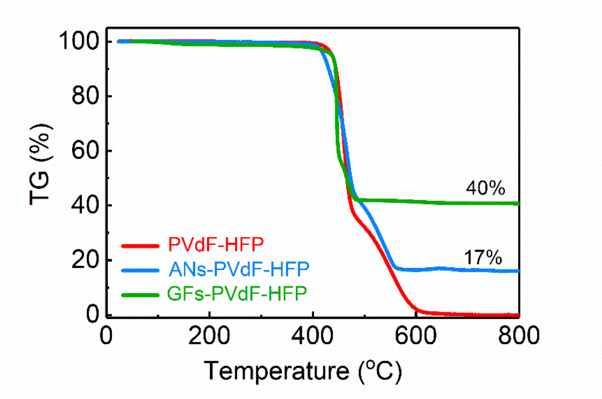

Supplementary Figure 7 TGA curves of PVdF-HFP, ANs-PVdF-HFP and GFs-PVdF-HFP membranes.

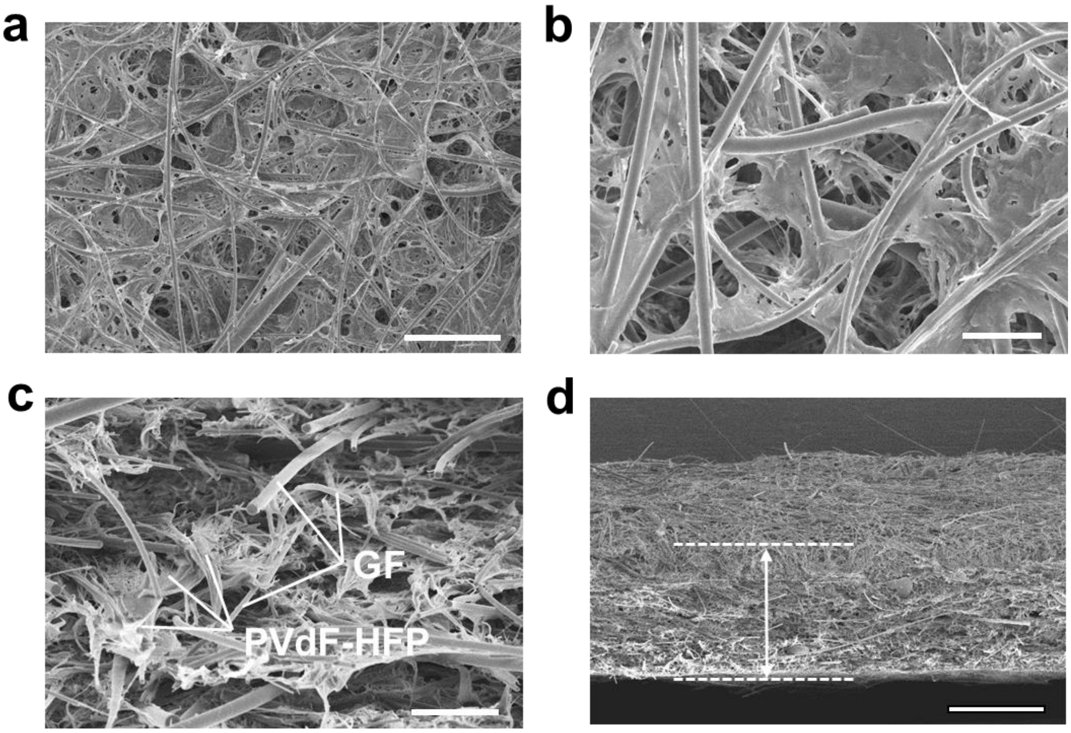

Supplementary Figure 8 SEM images of GFs-PVdF-HFP membranes. (**a**-**b**) top view, (**c**-**d**) cross section. Scale bars, 25 μm in **a**; 2 μm in **b**; 10 μm in **c**; 150 μm in **d**.

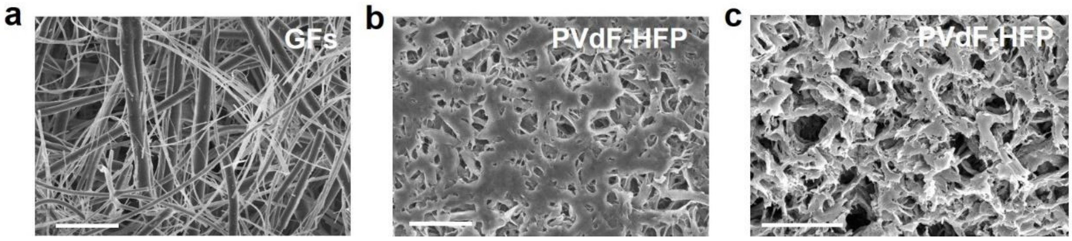

Supplementary Figure 9 SEM images of GFs and PVdF-HFP membranes. (**a**) top view of GFs, (**b**) top view and (**c**) cross section of PVdF-HFP. Scale bars, 5 μm in **a**, **b**, **c**.

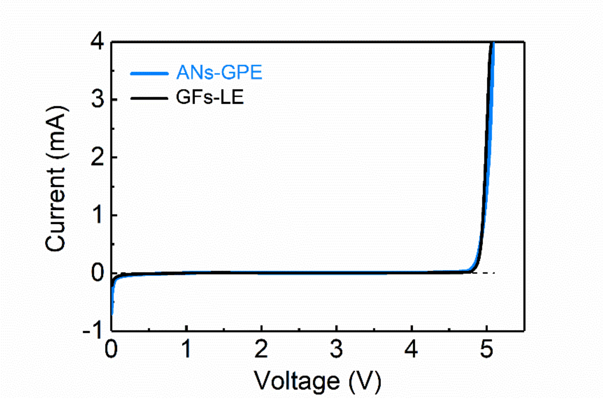

Supplementary Figure 10 Electrochemical stability window of GFs-LE and ANs-GPE determined by LSV at a scan rate of 1 mV s^{-1} with a stainless steel foil as the working electrode and a Na foil as the reference and counter electrode.

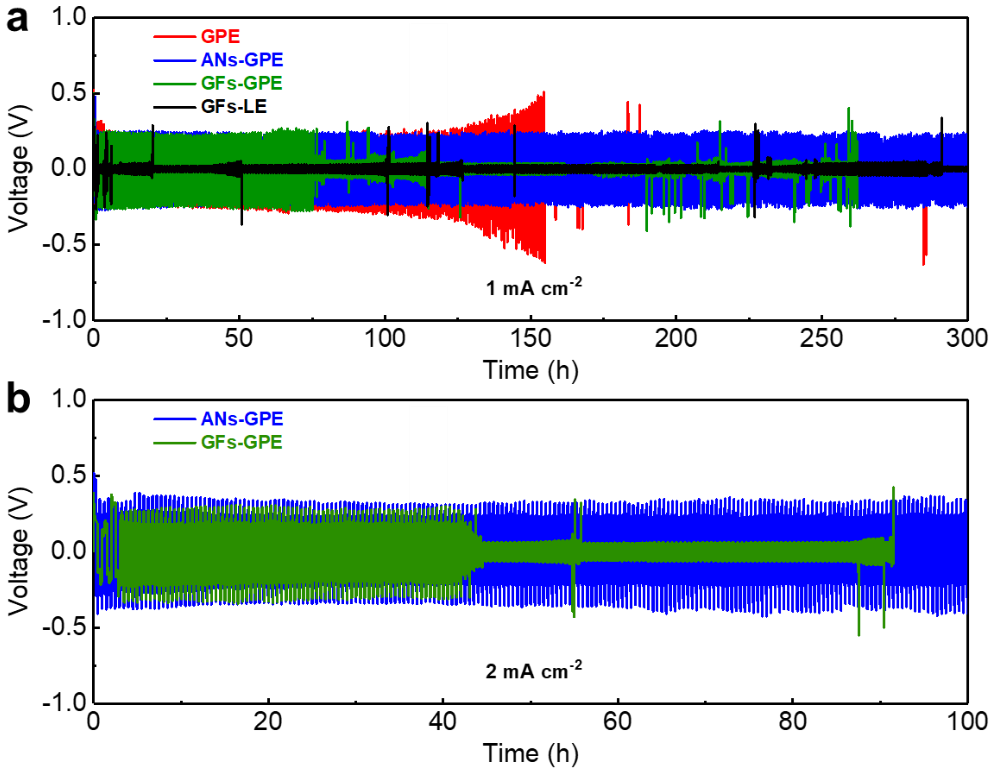

Supplementary Figure 11 Galvanostatic cycling curves of Na/Na symmetrical cells. (**a**) Na/Na symmetrical cells using GFs-LE, GPE, GFs-GPE and ANs-GPE at a current density of 1 mA cm^{-2}. (**b**) Na/Na symmetrical cells using GFs-GPE and ANs-GPE at a current density of 2 mA cm^{-2}.

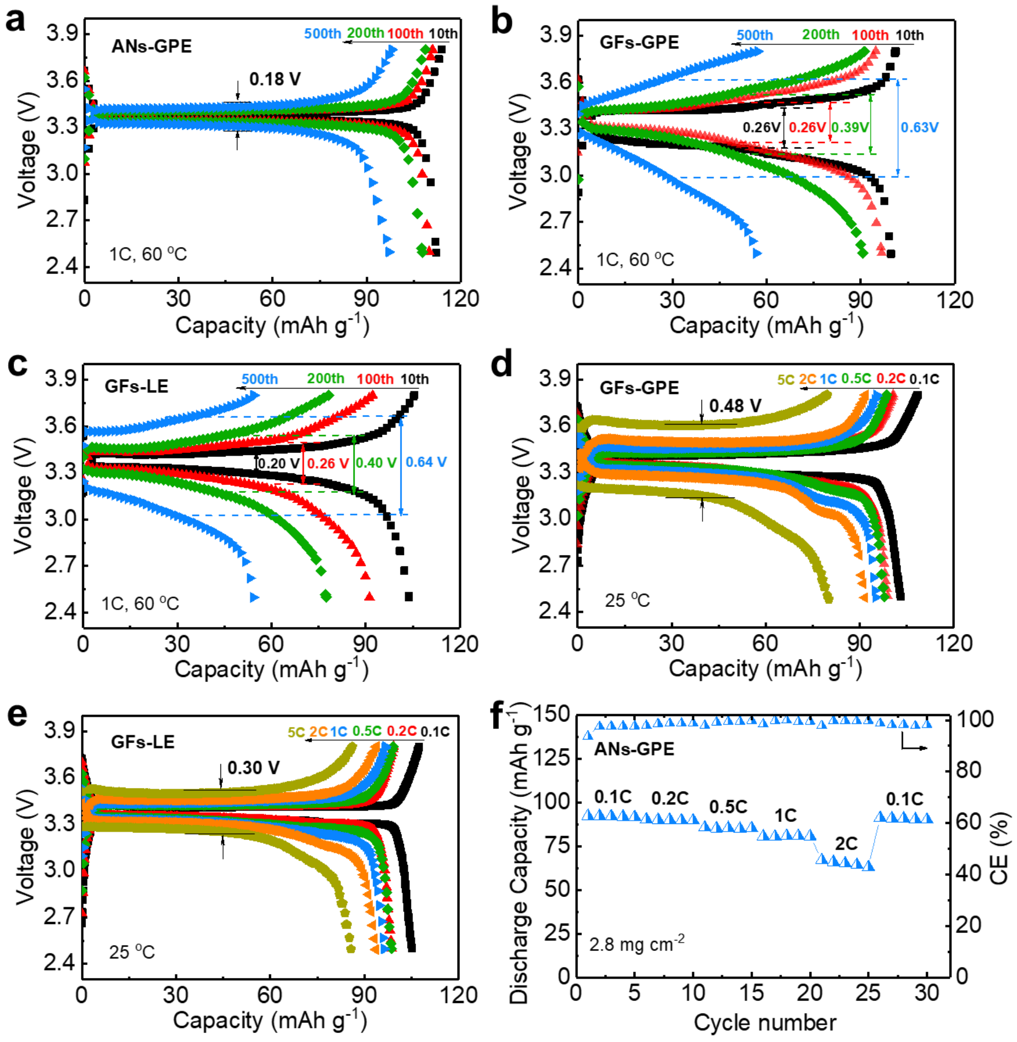

Supplementary Figure 12 Electrochemical performance of NVP/Na cells. (**a-e**) Charge-discharge curves of cells cycled at different times, temperatures and rates. (**f**) Rate performance of NVP/ANs-GPE/Na cell with NVP mass loading of 2.8 mg cm^{-2}.

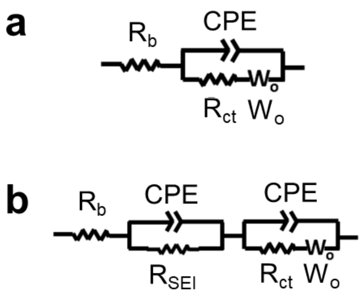

Supplementary Figure 13 Equivalent circuit models used for the EIS simulation.

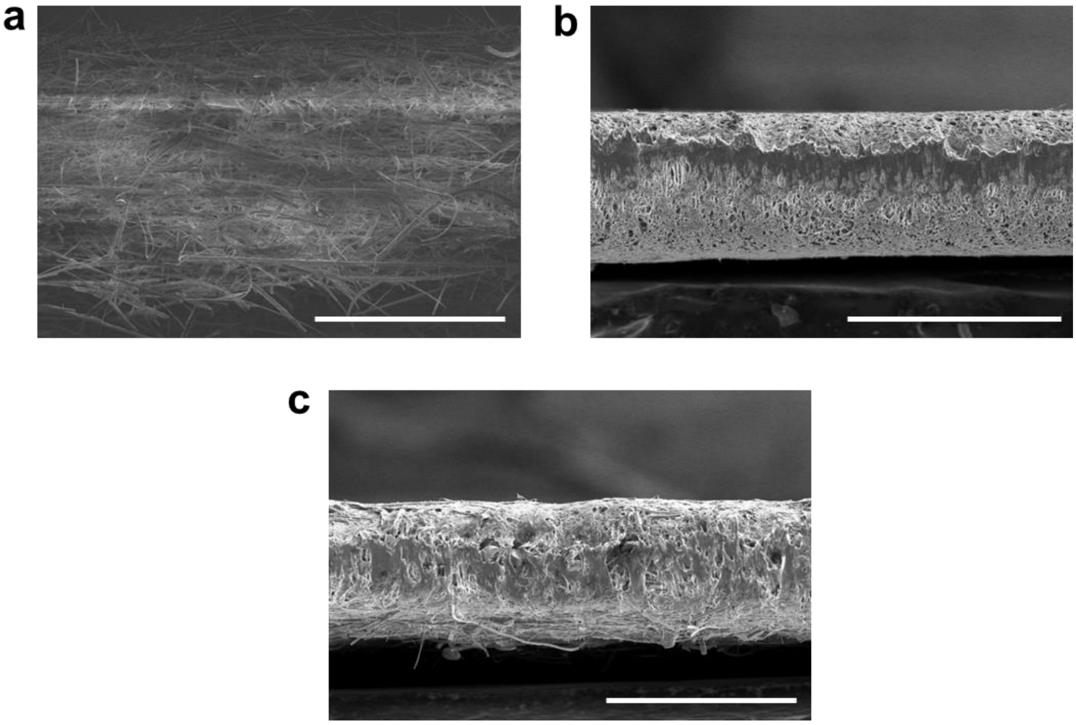

Figure 14 The cross-sectional SEM images of membranes. (**a**) 80-μm GFs, (**b**) 80-μm PVdF-HFP and (**c**) 80-μm GFs-PVdF-HFP. Scale bars, 100 μm in **a**, **b**, **c**.

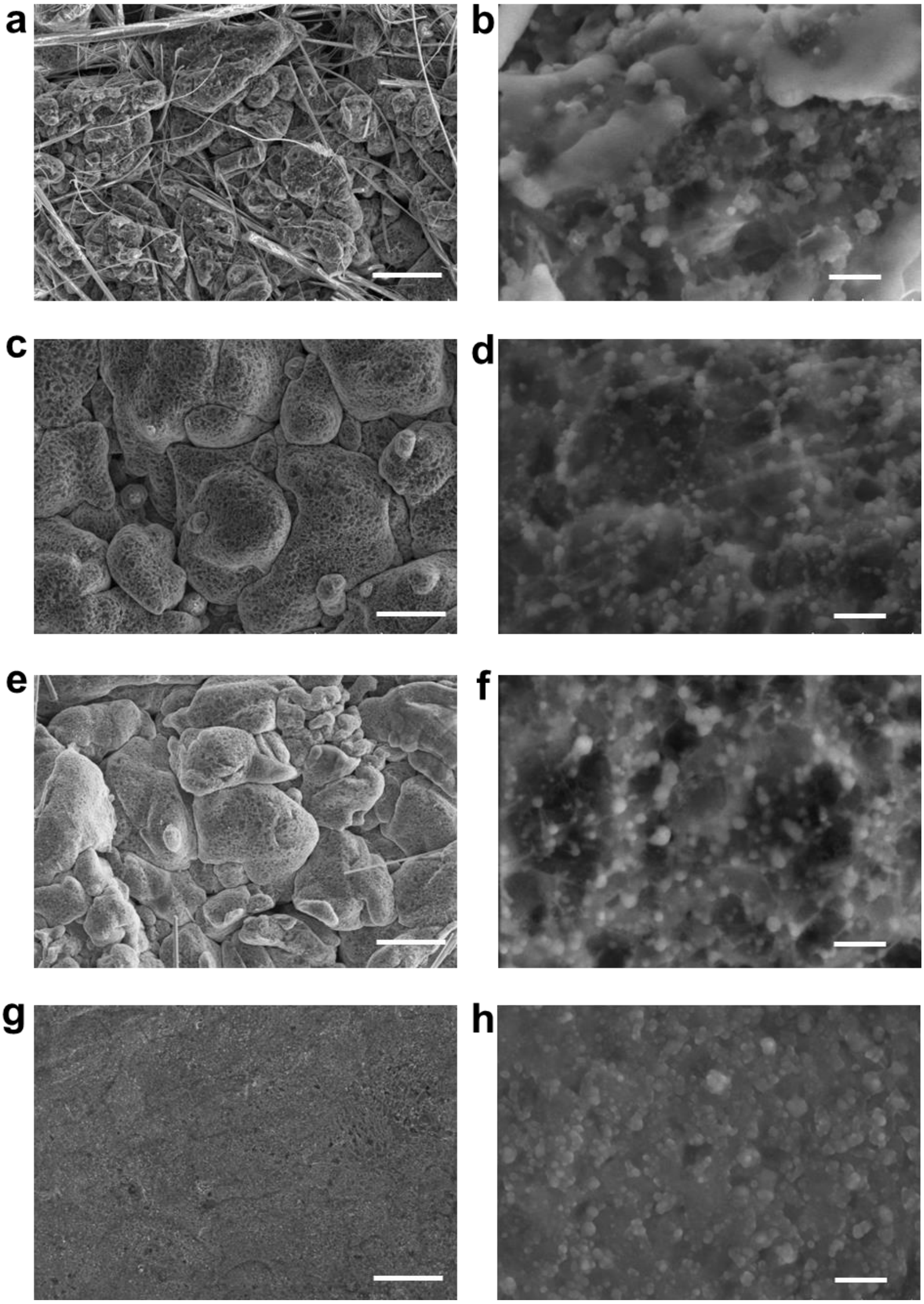

Supplementary Figure 15 SEM images of Cu electrodes from Cu/Na cells using different electrolytes. (**a**, **b**) GFs, (**c**, **d**) GPE, (**e**, **f**) GFs-GPE and (**g**, **h**) ANs-GPE with same liquid electrolyte uptake (40 μL) and thickness (80 μm). Areal capacity of 3 mAh cm^{-2} Na metal was deposited on the Cu surface. The SEM images of Supplementary Figure 15**b**, **d**, **f** and **h** are magnified images of Figure 15**a**, **c**, **e** and **g**. Scale bars, 10 μm in **a**, **c**, **e**, **g**; 0.5 μm in **b**, **d**, **f**, **h**.

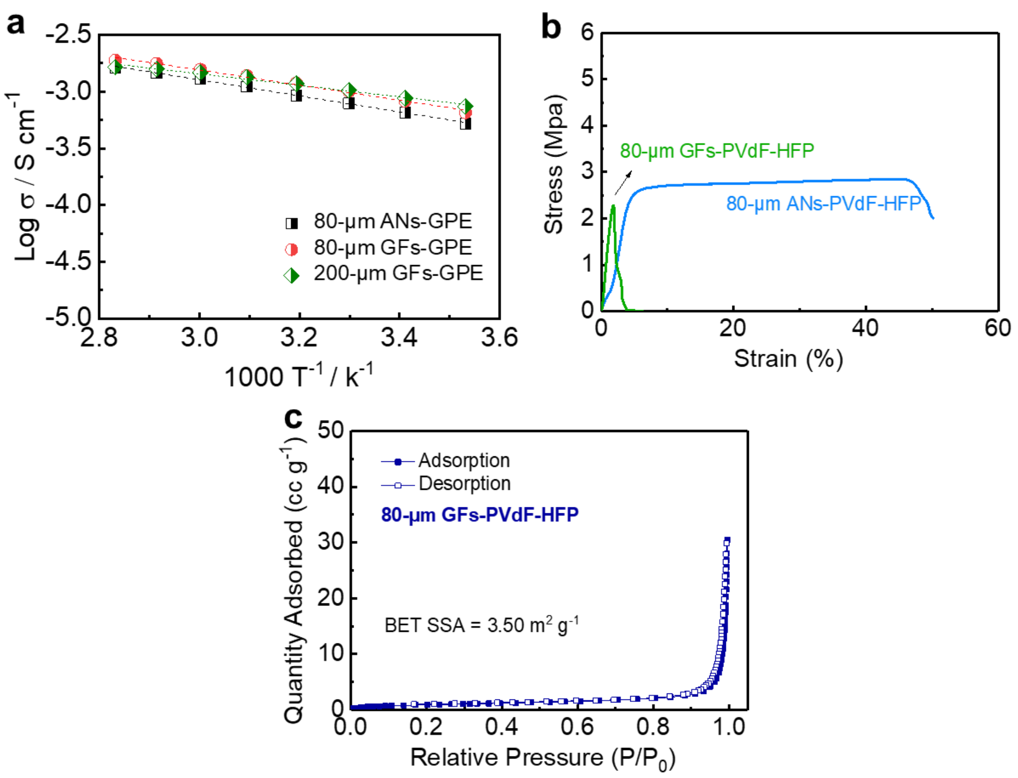

Supplementary Figure 16 Characterization of the physical properties of ANs-PVdF-HFP and GFs-PVdF-HFP. (**a**) Ionic conductivities of 80-μm ANs-GPE, 80-μm and 200-μm GFs-GPE, (**b**) stress-strain curves of 80-μm ANs-PVdF-HFP and 80-μm GFs-PVdF-HFP; (**c**) Nitrogen adsorption/desorption isotherms of 80-μm GFs-PVdF-HFP.

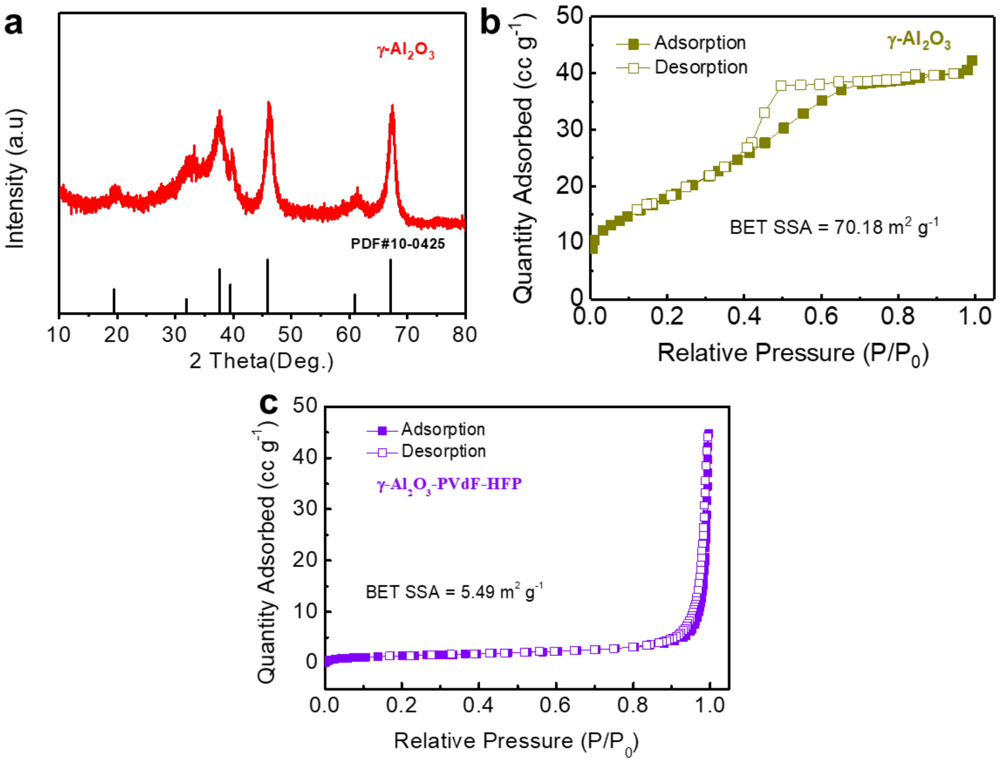

Supplementary Figure 17 XRD and BET characterization of γ-Al_2O_3 and γ-Al_2O_3-PVdF-HFP. (**a**) XRD patterns of cross-linked γ-Al_2O_3 nanowires, Nitrogen adsorption/desorption isotherms of (**b**) γ-Al_2O_3 and (**c**) γ-Al_2O_3-PVdF-HFP.

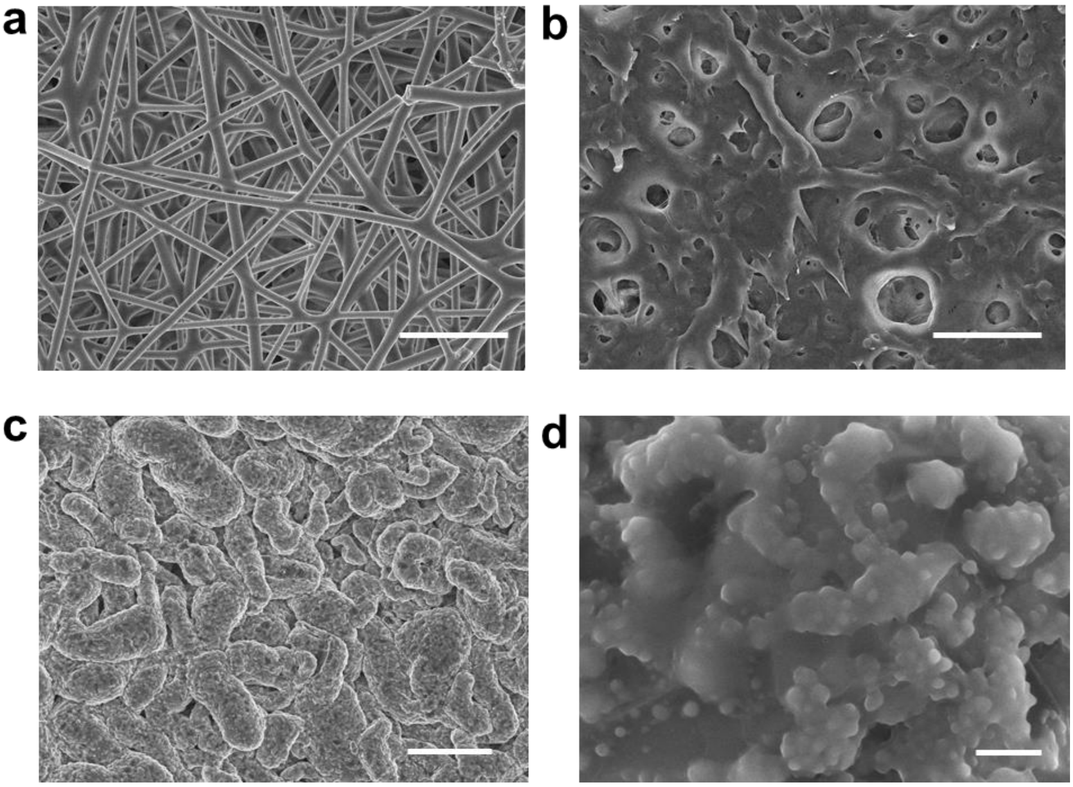

Supplementary Figure 18 SEM images of γ-Al_2O_3-based membranes and Cu electrode. (**a**) γ-Al_2O_3, **(b)** γ-Al_2O_3-PVdF-HFP, (**c**, **d**) Cu electrode from Cu/Na cell using γ-Al_2O_3-GPE, 3 mAh cm^{-2} areal capacity of Na metal was deposited on the Cu surface at current densities of 0.5 mA cm^{-2} and 60 ºC. Scale bars, 5 μm in **a, b**; 10 μm in **c**; 0.5 μm in **d**.

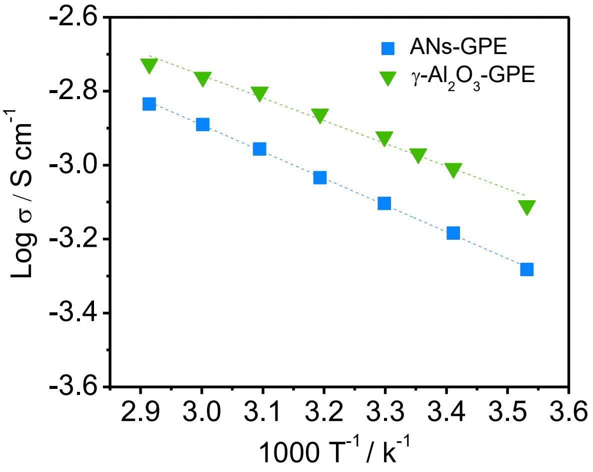

Supplementary Figure 19 Ionic conductivities of 80-μm ANs-GPE and 80-μm γ-Al_2O_3-GPE.

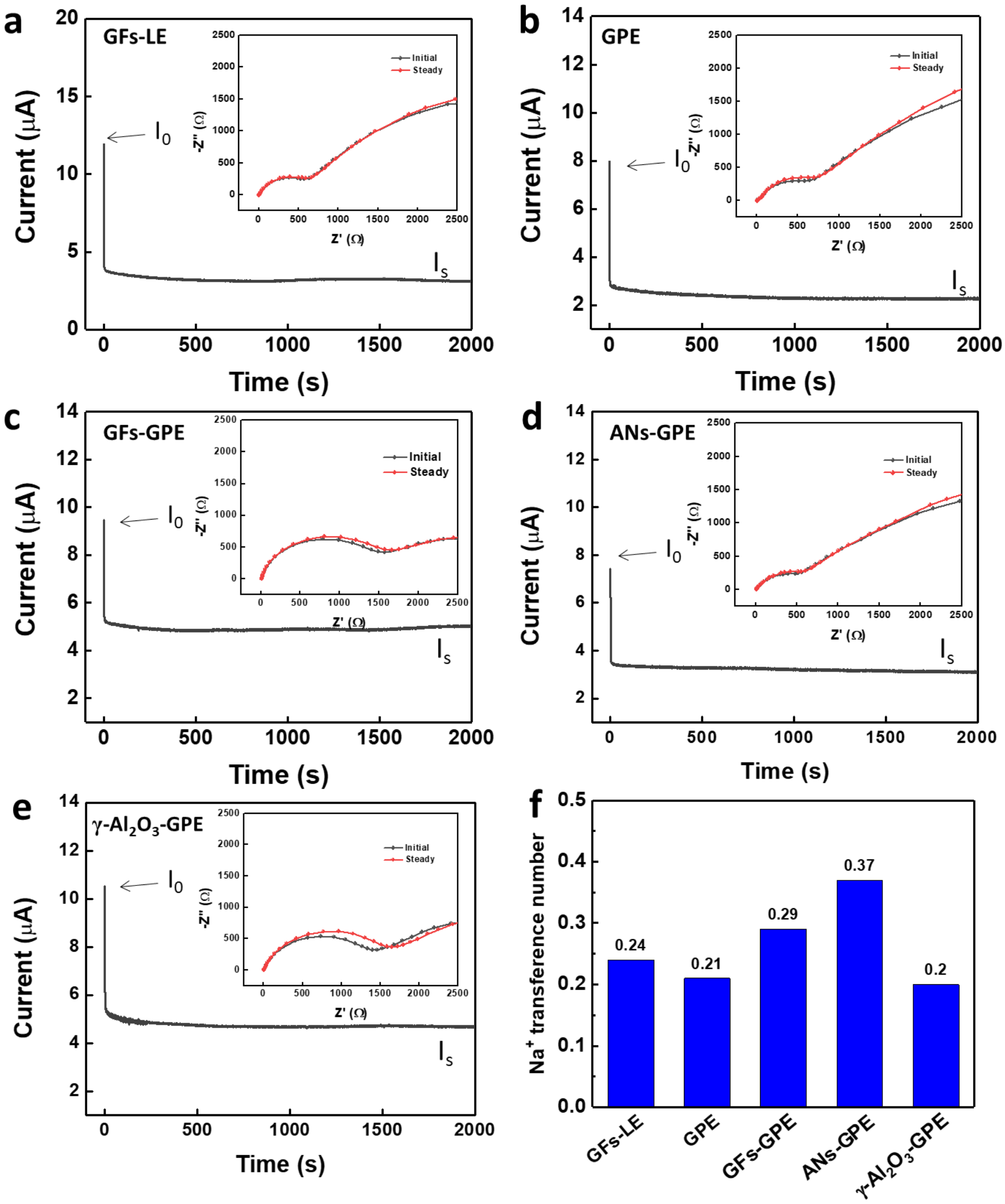

Supplementary Figure 20 Measurement of Na-ion transference number of different electrolytes. The chronoamperometry profiles of Na/Na symmetrical cells using (**a**) GFs-LE, (**b**) GPE, (**c**) GFs-GPE, (**d**) ANs-GPE and (**e**) γ-Al_2O_3-GPE under a polarization voltage of 20 mV, and the EISs before and after the polarization (see inset), (**f**) the comparison of Na-ion transference number of GFs-LE, GPE, GFs-GPE, ANs-GPE and γ-Al_2O_3-GPE.

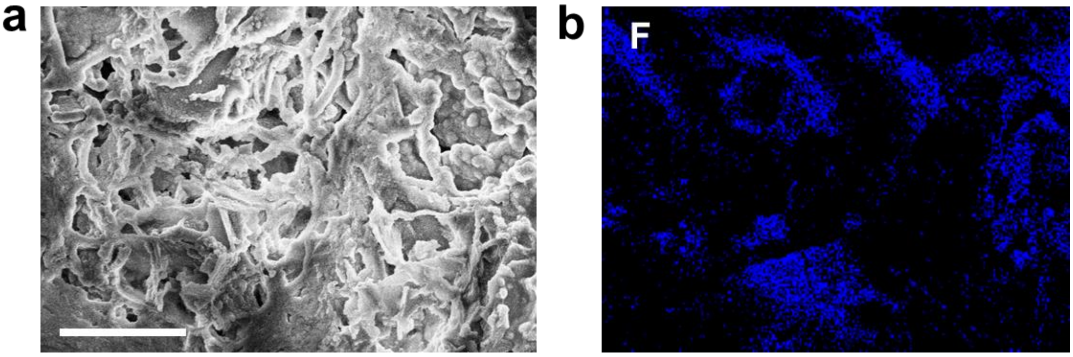

Supplementary Figure 21 Morphology and EDS Characterization of the Na anode. (**a**) SEM and (**b**) EDS images of the Na anodes after 200 cycles at 1C and 60 ºC using GFs-LE. Scale bars, 5 μm in **a**.

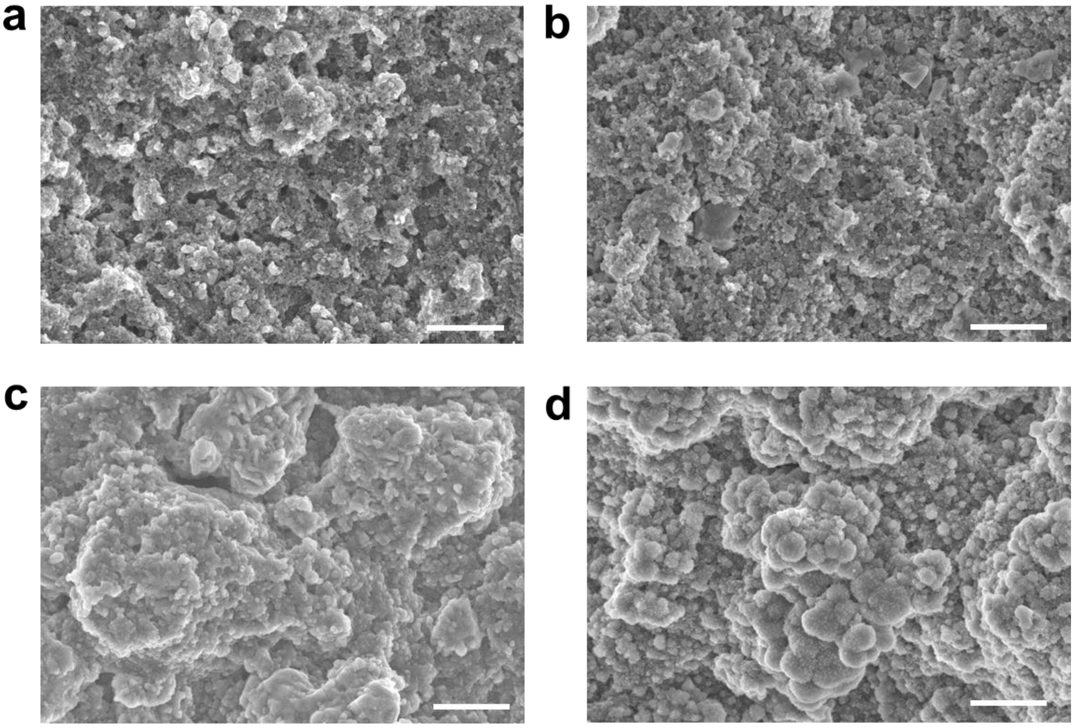

Supplementary Figure 22 SEM images of the NVP electrodes. Fresh NVP electrode (**a**) and NVP electrodes after 1000 cycles at 1C and 60 ºC with (**b**) ANs-GPE, (**c**) GFs-GPE, (**d**) GFs-LE. Scale bars, 2 μm in **a**, **b**, **c**, **d**.

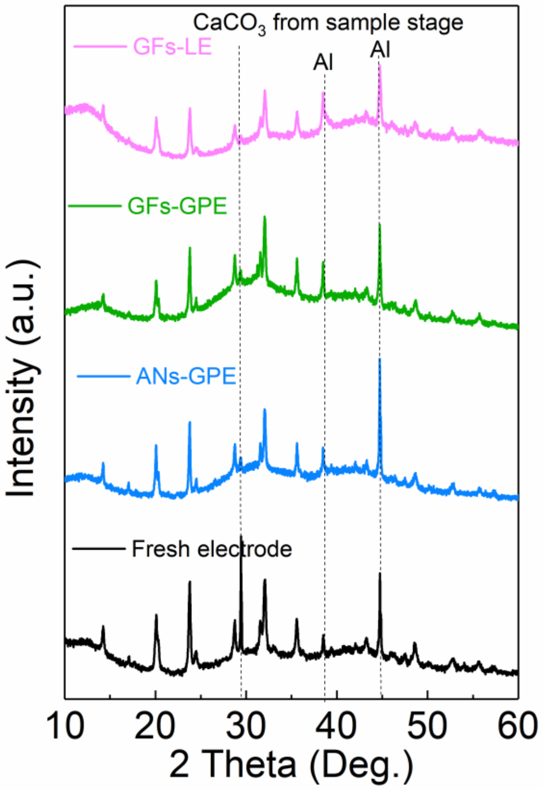

Supplementary Figure 23 XRD patterns of fresh NVP electrode and NVP electrodes after 1000 cycles at 1C and 60 ºC with GFs-LE, GFs-GPE and ANs-GPE.

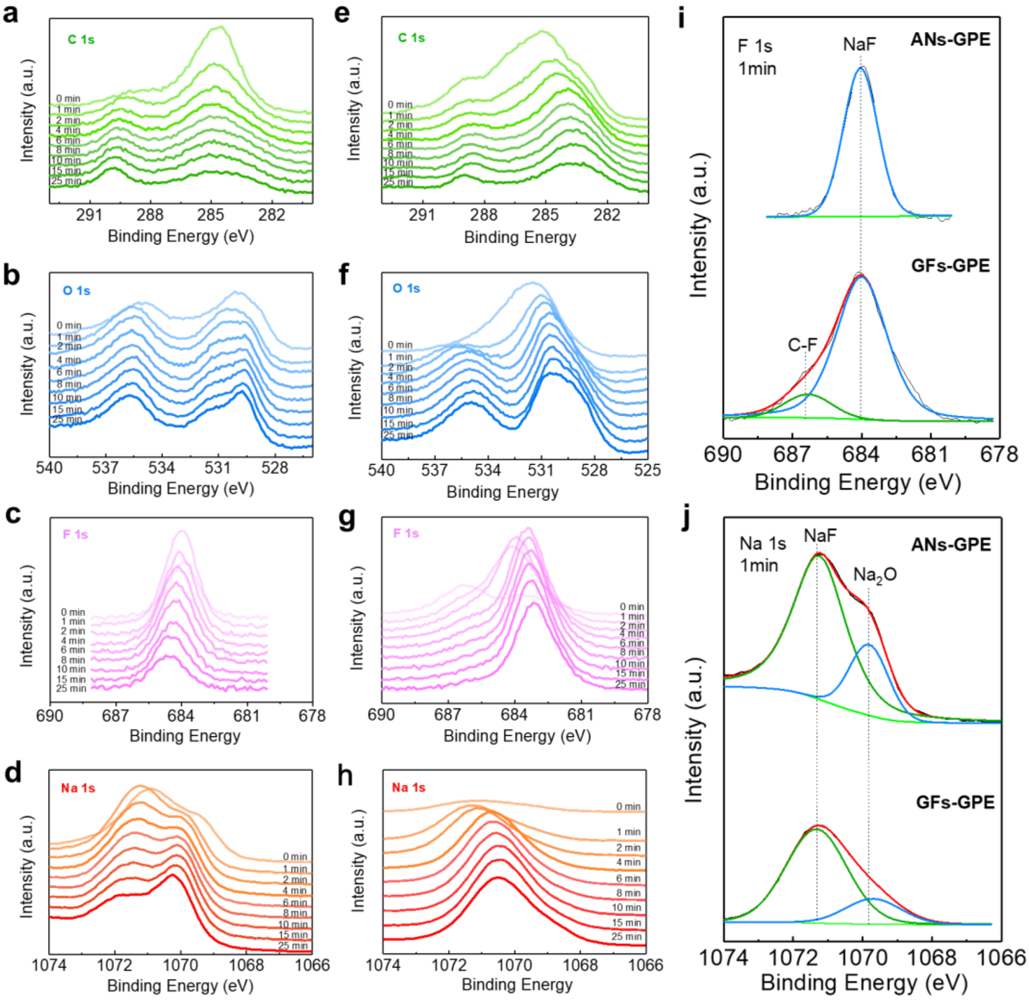

Supplementary Figure 24 XPS spectra of Na anodes after 200 cycles at 1C and 60 ºC. C1s, O1s, F1s and Na1s XPS spectra from of Na anodes from (**a-d**) NVP/ANs-GPE/Na cell and (**e-h**) NVP/GFs-GPE/Na cell after sputtering for different time. Fitted XPS spectra of (**i**) F1s and (**j**) Na1s of Na anodes from NVP/ANs-GPE/Na cell and NVP/GFs-GPE/Na cell.

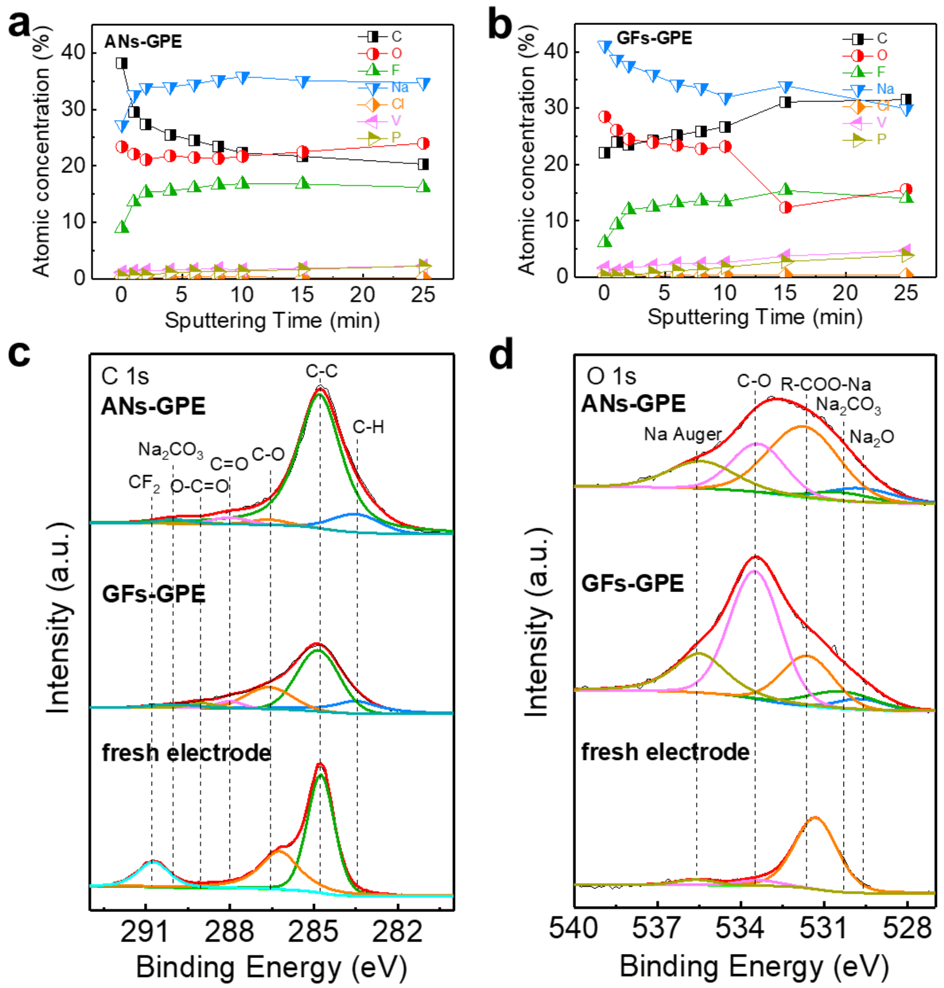

Supplementary Figure 25 XPS analysis of NVP electrodes. Atomic concentration of various elements with sputtering time from cells after 1000 cycles at 1C and 60 ºC with (**a**) ANs-GPE, (**b**) GFs-GPE. High-resolution C1s (**c**) and O1s (**d**) XPS spectra of fresh NVP electrodes and NVP electrodes after 1000 cycles at 1C and 60 ºC with ANs-GPE and GFs-GPE.

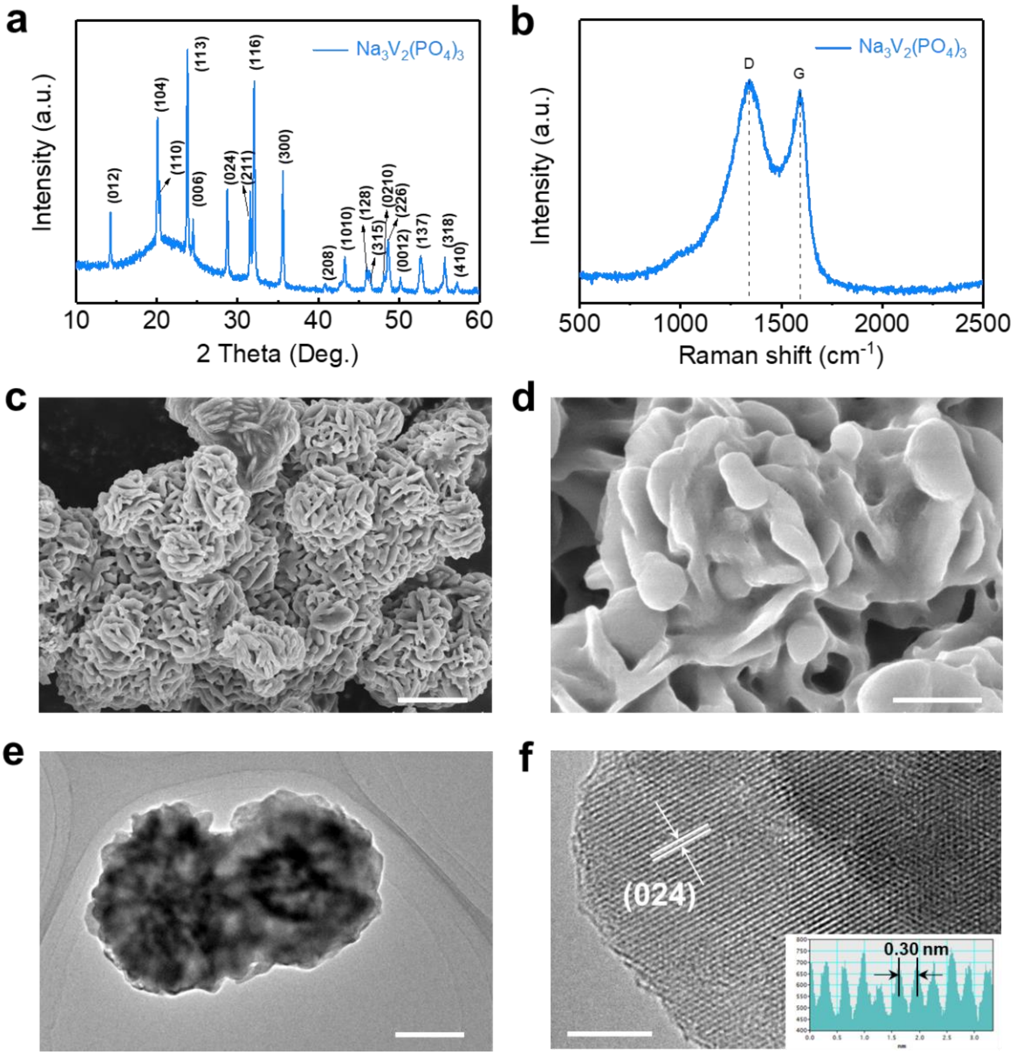

Supplementary Figure 26 Structure and morphology characterization of NVP particles. (**a**) XRD patterns, (**b**) Raman spectra, (**c-d**) SEM images, (**e-f**) HR-TEM (inset of (**f**) is the interplanar spacing). Scale bars, 2 μm in **c**; 500 nm in **d**, **e**; 5 nm in **f**.

Supplementary Tables

Supplementary Table 1 The binding energy of EC and DEC on the surface of the Si (001) and β″-Al_2O_3 (003) slabs.

Slabs	Binding energy (eV)	
	EC	DEC
β″-Al_2O_3 (003)	−0.730	−0.935
SiO_2(001)-H	−0.343	−0.401

Supplementary Table 2 The amount of liquid electrolyte uptake of different membranes.

Membranes	Before LE uptake (mg)	After LE uptake (mg)	Liquid electrolyte uptake (%)
GFs	14.9	149.3	900.6
PVdF-HFP	13.3	72.4	444.2
200-μm GFs-PVdF-HFP	31.1	146.8	371.9
80-μm GFs-PVdF-HFP	21.2	82.4	288.7
80-μm ANs-PVdF-HFP	11.5	50.5	339.5
γ-Al_2O_3-PVdF-HFP	14.1	70.2	397.2

Supplementary Notes

Supplementary Note 1

As shown in Supplementary Fig. 16b, the ANs-PVdF-HFP membrane with thickness of 80 μm has obviously larger tensile strength (2.84 MPa) and maximum strain(42.4%) than GFs-PVdF-HFP membrane with same thickness (1.83 MPa and 2.28%). This reinforced mechanical property of ANs-PVdF-HFP decreases the possibility of short circuit of Na metal batteries.

Supplementary Note 2

We also examined the BET surface area and the liquid electrolyte uptake rate of 80-μm GFs-PVdF-HFP membrane (Supplementary Figure 16c and Table 2). It is seen that the BET surface area of 80-μm GFs-PVdF-HFP membrane (3.50 m^2 g^{-1}) is similar with that of 200-μm GFs-PVdF-HFP membrane (3.78 m^2 g^{-1}), but is obviously larger than that of 80-μm ANs-PVdF-HFP (2.36 m^2 g^{-1}). Whereas, the liquid electrolyte uptake rate of 80-μm GFs-PVdF-HFP membrane (288.7%) is obviously less than that of ANs-PVdF-HFP (339.5%) due to the smaller adsorption energies of GFs for solvents such EC and DEC (Fig. 1b-c).

Supplementary Note 3

The γ-Al_2O_3-PVdF-HFP membrane shows different morphology compared to the ANs-PVdF-HFP membrane (Figure 2h and Supplementary Figure 18). We think that the difference in the morphology of above membranes results from the different surface area, pore volume and structure of ANs and γ-Al_2O_3. The surface area of γ-Al_2O_3 (70.18 m^2 g^{-1}) is almost 19 times higher than that of ANs (3.73 m^2 g^{-1}) and the pore volume of γ-Al_2O_3 (0.065 cm^3 g^{-1}) is almost 4 times higher than that of ANs (0.016 cm^3 g^{-1}) (Supplementary Figures 5 and 17). As a result, more PVdF-HFP and solvent (acetone and ethanol) were absorbed inside γ-Al_2O_3 in the dipping process. Therefore, a highly porous PVdF-HFP is formed inside cross-linked γ-Al_2O_3 nanowires compared to that in ANs, which results in a obviously higher BET surface area of γ-Al_2O_3-PVdF-HFP (5.29 m^2 g^{-1}) than ANs-PVdF-HFP (2.36 m^2 g^{-1}).

Supplementary Methods

Preparation of γ-Al_2O_3 nanowires membrane. The γ-Al_2O_3 nanowires membranes were synthesized using the same method as ANs. In a typical procedure, 8 mmol $Al(NO_3)_3 \cdot 9H_2O$ were dissolved in 7.6 mL deionized (DI) water by magnetic stirring at room temperature. Then, 2.4 mL HNO_3, 1.6 mL $C_2H_4O_2$ and 40 mmol $Al(C_3H_7OH)_3$ were added into the above solution and stirred for 24 h. After that, 0.3 g PVP was added and stirred for 12 h to obtain a homogeneous stable spinning sol. The γ-Al_2O_3 nanowires membrane precursors were obtained under the electrospinning voltage of 18 kV and pumping rate of 1 mL h^{-1}. At last, the above membranes were dried in a vacuum oven for 12 h and then heat treated with two steps (600 ºC for 2 h with the heating rate of 1 ºC min^{-1} and heat up to 800 ºC for 2 h with the heating rate of 5 ºC min^{-1}) in a muffle furnace to obtain the γ-Al_2O_3 nanowires membrane.

Electrochemical measurements. The Na-ion transference number (t_{Na^+}) of GFs-LE, GPE, GFs-GPE, ANs-GPE and γ-Al_2O_3-GPE are measured via the method proposed by Evans et al[1,2]. According to this technique, the fresh symmetric Na/Na cells using GFs-LE, GPE, GFs-GPE, ANs-GPE and γ-Al_2O_3-GPE were polarized by applying a voltage (ΔV=20 mV) for 2000 s. The initial and the steady-state values of current (I_0 and I_s) for the cells were recorded as shown in Supplementary Fig. 20. At the same time, the initial and steady-state values of interfacial resistance (R_0 and R_s) of Na metal/electrolyte interface before and after the polarization were obtained by testing the electrochemical impedance spectroscopy (EIS) of symmetric Na/Na cells from 5 MHz to 1 Hz with a 10 mV AC oscillation. The t_{Na^+} was calculated by using the following equation [1, 2]:

$$t_{Na^+} = \frac{I_s(\Delta V - I_0 R_0)}{I_0(\Delta V - I_s R_s)}$$

The inset figure in Supplementary Figure 20 is the EIS of symmetric Na/Na cells using GFs-LE, GPE, GFs-GPE, ANs-GPE and γ-Al_2O_3-GPE before and after polarization. The depressed semicircle observed at high and mid frequencies is attributed to the interfacial resistance (R) and constant phase elements (CPE) of Na metal/electrolyte interface. The values of R before (R_0) and after (R_s) the polarization are obtained by the simulating the EIS using Z-view software.

Supplementary References

1. Evans, J., Vincent, C. A., Bruce P. G. Electrochemical measurement of transference numbers in polymer electrolytes. *Polymer* **28**, 2324-2328 (1987).
2. Kumar, D., Hashmi, S. A. Ion transport and ion-filler-polymer interaction in poly(methylmethacrylate)-based, sodium ion conducting, gel polymer electrolytes dispersed with silica nanoparticles. *J. Power Sources.* **195**, 5101-5108 (2010).
